# Patient feasibility as a novel approach for integrating IRT and LCA statistical models into patient-centric qualitative data—a pilot study

**DOI:** 10.3389/fdgth.2024.1378497

**Published:** 2024-10-02

**Authors:** Matthias Klüglich, Bert Santy, Mihail Tanev, Kristian Hristov, Tsveta Mincheva

**Affiliations:** ^1^ Therapeutic Area Oncology Medicine, Boehringer Ingelheim International GmbH, Biberach, Germany; ^2^ Clinical Development and Operations, Boehringer Ingelheim SComm, Belgium, Germany; ^3^ Medical Advisory Department, FindMeCure Ltd., London, United Kingdom

**Keywords:** IRT, latent traits, LCA, patient-centric data, patient feasibility

## Abstract

**Introduction:**

Clinical research increasingly recognizes the role and value of patient-centric data incorporation in trial design, aiming for more relevant, feasible, and engaging studies for participating patients. Despite recognition, research on analytical models regarding qualitative patient data analysis has been insufficient.

**Aim:**

This pilot study aims to explore and demonstrate the analytical framework of the “patient feasibility” concept—a novel approach for integrating patient-centric data into clinical trial design using psychometric latent class analysis (LCA) and interval response theory (IRT) models.

**Methods:**

A qualitative survey was designed to capture the diverse experiences and attitudes of patients in an oncological indication. Results were subjected to content analysis and categorization as a preparatory phase of the study. The analytical phase further employed LCA and hybrid IRT models to discern distinct patient subgroups and characteristics related to patient feasibility.

**Results:**

LCA identified three latent classes each with distinct characteristics pertaining to a latent trait defined as patient feasibility. Covariate analyses further highlighted subgroup behaviors. In addition, IRT analyses using the two-parameter logistic model, generalized partial credit model, and nominal response model highlighted further distinct characteristics of the studied group. The results provided insights into perceived treatment challenges, logistic challenges, and limiting factors regarding the standard of care therapy and clinical trial attitudes.

## 1 Introduction

In recent years, the clinical research field has displayed a significant fundamental shift from a product-centric research and development paradigm to a more holistic and collaborative approach that engages patients and healthcare communities as co-creators and partners in drug development (1). This change reflects an ongoing effort to address delays, inefficiency, and the integration of the patient experience and perspective throughout the research process (2) and is defined broadly as “patient-centricity.”

Advancing this context, there is a growing body of evidence suggesting that actively involving patients in the design and conduct of clinical trials can lead to the identification of valuable insights that inform further trial development and may potentially improve patient recruitment and retention (3–5). Furthermore, efforts in understanding and integrating patient perspectives can help develop patient-focused adaptations in trial protocols (6).

Shifting from conceptual to practical terms, one area of the clinical research process that could also benefit from patient-centric efforts is the so-called feasibility assessment. This process involves assessing various factors to determine the practicality and viability of conducting a clinical trial. These assessments are crucial in preventing potential premature trial discontinuations, protocol amendments, and other events resulting in a significant waste of resources (7). In addition, sponsors are increasingly focusing on real-world data to inform data-driven decisions regarding different components of a planned trial, such as eligibility criteria evaluation (8). Although a complex concept with many variables, in this process, observational patient-derived data can play an important role in informing more effective trial protocols to achieve objectives (9).

Based on the presented context, this pilot study employs two statistical models in a novel analytical framework called “patient feasibility.” This framework is a two-step process for the evaluation and quantification of selected patient experiences in various aspects of the standard of care treatment.

A patient’s perspective of the standard of care treatment is a valuable tool for assessing the quality of care (10) and identifying key elements that ultimately impact the perceived patient-centricity of an intervention or a treatment regimen (11).

In this context, the standard of care insights surfaced by the “patient feasibility” framework can then be used to inform different parts of a trial design to achieve a more patient-tailored experience within a clinical trial. The proposed analytical framework in this study achieves this through two subsequent methods—latent class analysis (LCA) and interval response theory (IRT) models.

LCA is a person-centered categorical data analysis method that identifies latent classes based on similar response patterns within groups of surveyed people (12). On the other hand, IRT models present a statistical method that explores the relationship between individuals’ latent traits and their responses to items in a questionnaire (11). However, a limitation of the LCA is the potential struggle to capture the continuous nature of patient latent traits (12), and IRT may not fully account for the complex relationships between latent traits and item responses (13).

To overcome the mentioned limitations, the combined use of LCA and IRT has shown promise in some studies. For instance, Wu et al. applied IRT and LCA to examine comorbid substance use disorders in opioid-dependent patients, demonstrating the utility of these methods in understanding the heterogeneity of patient conditions (14). Similarly, Ueckert et al. explored the use of IRT in analyzing Alzheimer's Disease Assessment Scale - Cognitive Subscale (ADAS-Cog) data in Alzheimer's disease trials, showcasing the potential of combining pharmacometric modeling with IRT to enhance data analysis (15). However, LCA and IRT in both studies focused on a specific population, which may limit the broader applicability of the results to other patient populations or settings.

With this pilot study, the authors aim to test and expand the use of both mentioned statistical approaches in the context of understanding broader patient group heterogeneity and identifying latent traits within the standard of care context.

It has been previously noted that by integrating patient perspectives gained from standard of care therapy, clinical trials can be customized to better accommodate patient needs thus increasing patient-centricity in clinical research (16).

The presented exploratory study highlights the joint efforts between Boehringer Ingelheim and FindMeCure in understanding and quantifying the patient experience across three key latent traits—logistic challenges, healthcare engagement, and disease burden on daily life. The name is derived from traditional feasibility processes undertaken in clinical trial planning such as country and site selection. With patient feasibility, the authors aim to develop future machine learning (ML) analytical frameworks that can explore meaningful patient-reported outcomes and surface insights from various treatment settings.

## 2 Aim

The aim of this pilot study was to demonstrate the basic analytical framework of a novel approach for the utilization and integration of patient-centric data called “patient feasibility” through IRT and LCA analysis.

## 3 Data and methodology

### 3.1 Patient survey

This study involved patients diagnosed with glioblastoma (an aggressive type of brain cancer) from the USA and Germany in the standard of care setting. Participants included a diverse demographic without restrictions on age or sex, chosen to capture varied patient experiences due to the contrasting healthcare systems of these two countries.

The dataset was created from a 16-question survey with open-ended questions (also called items later in the presentation). Each question is associated with one of the three predefined themes examining different aspects and stages of the patient journey under the standard of care treatment, called latent traits: (1) healthcare engagement, (2) logistical challenges, and (3) disease impact on daily life. Latent traits are not directly measurable but are inferred from observable responses to the survey questions.

The aim was to understand how these latent traits and factors influence patient experiences and how the insights surfaced from LCA and IRT can be applied to a future clinical trial's patient-centricity strategy. The survey data were anonymized prior to analysis. The survey was conducted online and distributed through the FindMeCure (FindMeCure Ltd.) platform to target population groups from Germany and the USA. In total, the survey captured responses from 113 patients.

### 3.2 Measurements

The study uses content analysis to transform survey answers into discrete observations that can be used in statistical models. Qualitative content analysis of the free-form responses was used to understand the nuances and repeating patterns. The most meaningful themes that best represent the data are systematically categorized and assigned a numerical code.

Depending on the type of question, the categorized data represent the following measurement levels:

• Nominal—distinct categories, without an inherent ranking, relationship, or hierarchy between the elements;
• Ordinal—categories have a meaningful ranking but the interval between the elements is not necessarily equal; and
• Dichotomous—this is a type of nominal data that represents a binary choice, in the current case, yes/no questions and the region.

Table 1 outlines the qualitative content analysis.

**Table 1:** Content analysis and coding.

Item No.	Survey question (item)	Category label	Category	Values	Measure
1	Select country	Region	Region	1- Germany 2- USA	Nominal (D)
2	Have you received therapy yet?	Received therapy	Rcvd_Therapy	Yes—1 No—0	Nominal (D)
3	What is the received therapy type?	Therapy type	Therapy_Type	1- Antibody 2- No therapy yes 3- TTF device 4- Radiotherapy 5- Surgery 6- Surgery + another therapy 7- Chemotherapy 8- Chemotherapy + another therapy	Nominal
4	What is your motivation for joining a clinical trial?	Motivation	Motivation	1- Cure 2- Survival 3- Quality of life	Nominal
5	How much time have you discuss treatment options with your physician?	Time to discuss treatment	Treat_Discuss_Time	1- Less than 1 day 2- 1 day–1 month 3- Over 1 month	Ordinal
6	How many treatment appointments do you have per month?	Appointments per month	Appt_Count	1- 0–2 2- 2 to 5 3- More than 5	Ordinal
7	What is your treatment commute time?	Commute	Commute	1- 0–30 min 2- 30–60 min 3- ≥60+ min	Ordinal
8	What is the average appointment duration?	Appointment time	Appt_Time	1- 0–60 min 2- 60–120 min 3- 120+ min	Ordinal
9	What part of your appointment do you dislike?	Appointment dislike part	Appt_Dislike	1- None 2- Treatment related 3- Organization related	Nominal
10	Did your physician offer you to join a clinical trial?	Clinical trial offered	Offered_Trial	1- Yes 2- No	Nominal (D)
11	What financial impact has the treatment on your life?	Financial impact	Fin_Impact	1- None 2- Moderate 3- Heavy	Ordinal
12	Have you experienced side effects from the current treatment?	Side effects	Side_Effects	1- Yes 2- No	Nominal (D)
13	Were you taken off treatment due to side effects?	Taken off treatment	Off_Treatment	1- Yes 2- No	Nominal (D)
14	What treatment options were offered for the side effects?	Off-treatment options	Off_Treat_Options	1- None 2- SoC 3- Another therapy	Nominal
15	Is there evidence of disease progression?	Disease progression	Disease_Prog	Yes—1 No—0	Nominal (D)
16	What treatment options were offered for disease progression?	Disease progression treatment	Progress_Treatment	1- Another therapy 2- SoC 3- No options 4- No Disease Progression	Nominal

For the IRT model, the binary measurements were coded with 1—yes, 0—no.

An important note within the context of the study survey is the item “Have you experienced side effects from the current treatment?” (Side_Effects). This item does not signify side effects in a strictly medical context but rather any subjective events related to a current treatment that the patient classifies as unwanted or negative.

There are 113 cases and 17 variables (see Supplementary Material S1 for measurement frequencies).

### 3.3 Methodology

The empirical analysis of the study employs a combination of LCA and hybrid IRT.

#### 3.3.1 Latent class analysis

The LCA is particularly useful when dealing with categorical patient data because it can reveal distinct groups of patients (latent classes) that have similar treatment experiences with a comparable disease impact on their daily life.

Each class is identified based on a combination of characteristics with conditional probabilities, showing how likely it is for a variable to take certain values. The latent class must be solved for statistically independent variables to ensure that the identified subgroups are characterized only by the patterns of association.

The latent class model form is shown in Equation 1 (LCA model form):

(1) $$p_{i_{1},i_{2},\ldots ,i_{N}}\approx \sum_{t}^{T}\;p_{t}\prod_{n}^{N}\;p_{i_{n},t}^{n}$$

where *T* denotes the number of latent classes, *p_t_* is the unconditional probability (total sum to 1), and $p_{i_{n},t}^{n}$ is the conditional probability.

#### 3.3.2 IRT model

IRT is a statistical framework that can provide deeper insights into the patient-reported outcomes. The models in this family can indicate how individual items, in this case patient responses to survey questions, are associated with a latent trait.

##### 3.3.2.1 Two-parameter logistic model for dichotomous items

The two-parameter logistic (2PL) model is commonly employed for dichotomous items. The probability of a correct response $P(X_{ij}=1)$ for person *i* on item *j* in a 2PL model is given by Equation 2 (2PL model form):

(2) $$P(X_{ij}=1)=\frac{1}{1+\exp\;\left(-a_{j}(\theta_{i}-b_{j})\right)}$$

where $P(X_{ij}=1)$ is the probability of a correct response, $a_{j}$ is the discrimination parameter for item *j*, $\theta_{i}$ is the latent trait for person *i*, $b_{j}$ is the difficulty parameter for item *j*, and exp is the exponential function.

##### 3.3.2.2 The graded partial credit model for ordinal items

The graded partial credit model (GPCM) is used for ordinal variables. The probability of observing a response category *k* or less for person *i* on item *j* in a GPCM is given by Equation 3 (GPCM model form):

(3) $$P(X_{ij}\leq k)=\frac{\exp\;\left(\sum_{m=1}^{k}\;a_{\;jm}(\theta_{i}-b_{\;jm})\right)}{1+\sum_{m=1}^{K}\;\exp\;\left(\sum_{m=1}^{K}\;a_{\;jm}(\theta_{i}-b_{\;jm})\right)}$$

where $P(X_{ij}\leq k)$ is the probability of observing response category *k* or less, $a_{\;jm}$ is the discrimination parameter for category *m* of item *j*, $b_{\;jm}$ is the difficulty parameter for category *m* of item *j*, and *K* is the total number of response categories for the ordinal item.

##### 3.3.2.3 Nominal response model for nominal items

The nominal response model (NRM) is used for the nominal measurements. The probability of observing a response in category *k* for person *i* on item *j* in an NRM is given by Equation 4 (NRM model form):

(4) $$P(X_{ni}=k)=\frac{\exp\;(\alpha_{ik}\cdot \theta_{n}-\beta_{ik})}{\sum_{\;j=1}^{J}\exp\;(\alpha_{ij}\cdot \theta_{n}-\beta_{ij})}$$

where $P(X_{ij}=k)$ is the probability of observing response category *k* or less, $a_{\;jm}$ is the discrimination parameter for category *m* of item *j*, $b_{\;jm}$ is the difficulty parameter for category *m* of item *j*, and *K* is the total number of response categories for the ordinal item.

LCA was performed in SPSS (Version 29, IBM, Chicago, IL, USA). IRT models were performed in Stata 14 software (2015, StatCorp, College Station, TX, USA).

#### 3.3.3 The combination of LCA and IRT

First, LCA is used to identify subgroups of patients who respond similarly to survey items. This allows the segmentation of the patient population into meaningful clusters based on their response patterns. Second, the IRT model is fitted for the whole dataset and difficulty and discrimination parameters are estimated. Subsequently, the distribution of the latent traits is examined across the identified latent classes and then the overall item parameters are analyzed for significant variances in the observed response patterns of different latent classes.

## 4 Results

The basic identified latent traits for all used analyses can be categorized into the following three groups:

• Disease impact on daily life—understand how a patient’s medical condition and assigned treatment affect their life through the presence of side effects, disease progression, dissatisfying treatment aspects, and financial impact.
• Healthcare engagement—explore patterns in the time patients spend with the physician to discuss treatment, the physician’s awareness of clinical trials, the patient’s motivation to join a clinical trial, and options for alternative therapy.
• Logistical challenges**—**identify challenges that patients face in the course of their treatment, including the burden from multiple appointments per month, long appointment durations, and long commute time.

### 4.1 LCA

**Figure 1:**
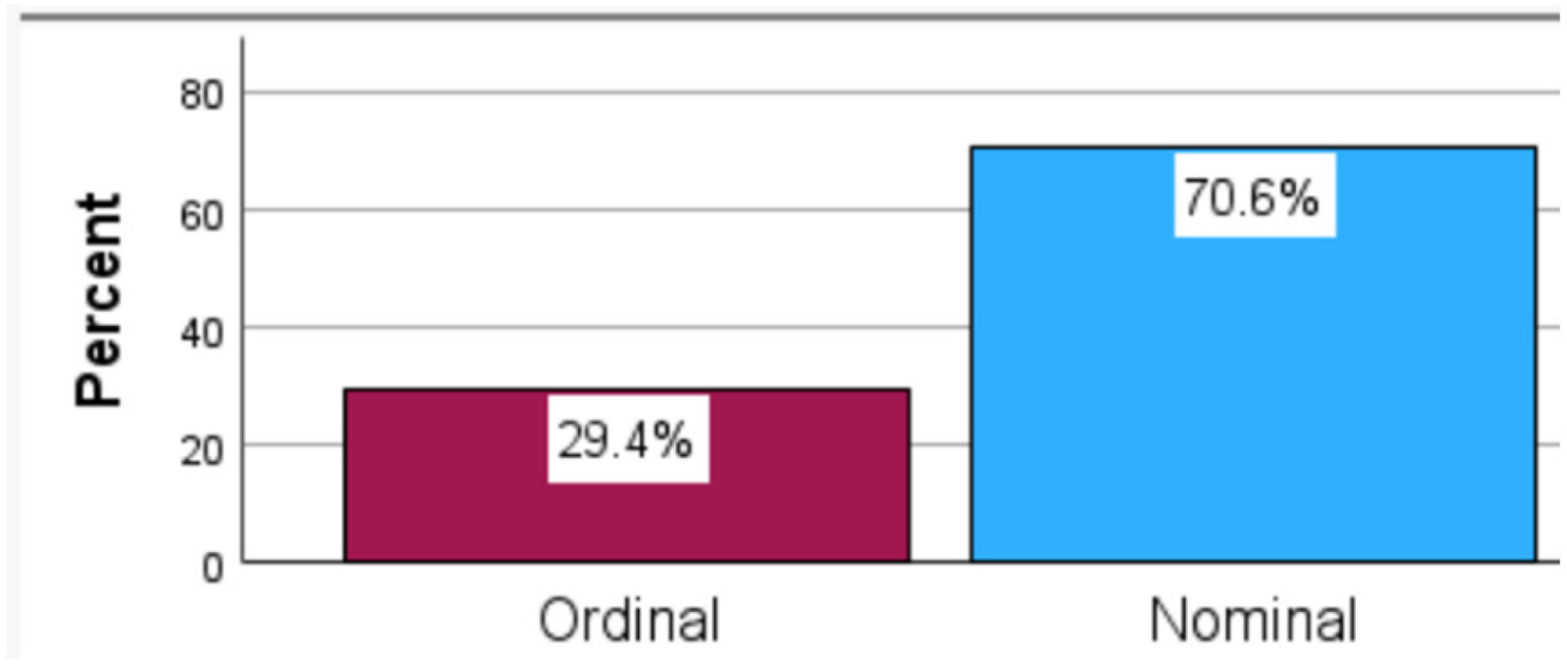
Measurement levels (source: SPSS).

The LCA is employed to uncover the distinct patient subgroups and reveal patterns of associations (latent classes) within the observed variables (Figure 1). The best model fit was identified by comparing five models (iterations) with different numbers of latent classes and combinations of variables and covariates, selecting the one with the lowest Akaike information criterion (AIC).

The AIC is utilized in the LCA to aid in model selection and determine the optimal number of classes within the analysis (17). By evaluating the AIC values of LCA models with different pre-selected numbers of classes, it is possible to identify the class number model with the lowest AIC as the most suitable choice, indicating a better fit (18) (Table 2).

**Table 2:** Model fit comparison.

Iteration	Variables	Covariates	No. of classes	AIC
1	Region, Rcvd_Therapy, Therapy_Type, Offered_Trial, Motivation, Appt_Dislike, Side_Effects, Progress_Treatment, Appt_Count, Fin_Impact, Commute, Treat_Discuss_Time, Off_Treatment, Off_Treatment_Options, Disease_Progression	Appt_Time	4	2,450
2	Region, Rcvd_Therapy, Therapy_Type, Offered_Trial, Motivation, Appt_Dislike, Side_Effects, Appt_Time, Fin_Impact, Commute, Treat_Discuss_Time, Off_Treatment, Off_Treatment_Options, Disease_Progression	Treat_Discuss, Appt_Count	4	2,325
3	Region, Therapy_Type, Offered_Trial, Motivation, Appt_Dislike, Side_Effects, Progress_Treatment, Treat_Discuss_Time, Appt_Count, Fin_Impact_Commute	Fin_Impact, Appt_Time	3	2,223
4	Region, Therapy_Type, Offered_Trial, Motivation, Appt_Dislike, Side_Effects, Progress_Treatment, Treat_Discuss_Time, Appt_Count, Fin_Impact	Commute, Appt_Time	3	2,066
5	Region, Therapy_Type, Offered_Trial, Motivation, Appt_Dislike, Side_Effects, Progress_Treatment, Treat_Discuss_Time, Appt_Count, Off_Treatment_Options	Commute, Appt_Time, Fin_Impact	3	2,178

**Figure 2:**
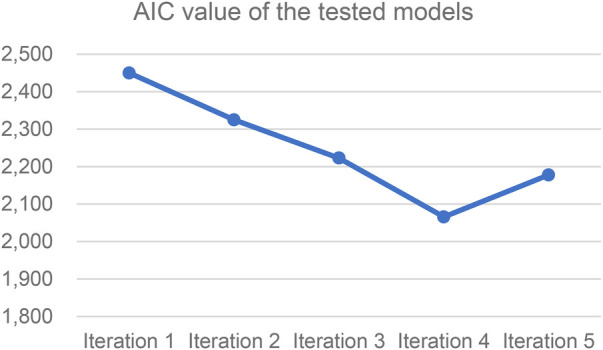
LCA model iteration values of the AIC.

The AIC provides balance between the model goodness-of-fit to the observed data and complexity. Lower values indicate a better fit. The analysis suggests that including more variables and classes does not lead to a better fit. This could be the result from a higher correlation between some survey questions, thus including them introduces higher model complexity but not a better fit of the underlying structure. Another reason could be that items are not equally informative and might not be relevant to the latent traits. The second part of the analysis, in which the IRT model is introduced, aims to address these questions by evaluating the quality and informative power of the response items and interpreting the results in the context of the identified latent classes. Figure 2 shows a comparison of AIC values of the five performed iterations.

The combination of variables and covariates (shown in Table 3) in the model from iteration 4 is used as the best fit as measured by the AIC. Table 3 presents the results from the latent class analysis for the selected model from iteration 4.

**Table 3:** LCA, latent class probabilities (source: SPSS).

Variable	Class	Estimated class conditional probabilities							
		1	2	3	4	5	6	7	8
Region	1	**0** **.** **732**	0.268	—	—	—	—	—	—
	2	0.333	**0**.**667**	—	—	—	—	—	—
	3	0.462	**0**.**538**	—	—	—	—	—	—
Therapy type	1	—	0.099	—	0.135	0.197	—	—	**0.569**
	2	—	1.000	—	—	—	—	—	—
	3	0.028	—	0.014	0.018	—	0.237	0.056	**0.648**
Offered trial	1	0.068	**0**.**932**	—	—	—	—	—	—
	2	0.238	**0**.**762**	—	—	—	—	—	—
	3	**0**.**525**	0.475	—	—	—	—	—	—
Motivation	1	**0**.**447**	0.302	0.201	0.050	—	—	—	—
	2	**0**.**524**	0.286	0.190	—	—	—	—	—
	3	**0**.**445**	0.263	0.236	0.056	—	—	—	—
Appointment dislike part	1	0.322	0.069	**0.610**	—	—	—	—	—
	2	**0**.**476**	0.333	0.190	—	—	—	—	—
	3	0.146	**0**.**524**	0.330	—	—	—	—	—
Side effects	1	0.154	**0**.**846**	—	—	—	—	—	—
	2	0.000	**1**.**000**	—	—	—	—	—	—
	3	**0**.**821**	0.179	—	—	—	—	—	—
Disease progress treatment	1	0.086	0.417	—	**0.497**	—	—	—	—
	2	—	—	0.274	**0.726**	—	—	—	—
	3	0.101	0.314	0.153	**0.431**	—	—	—	—
Time to discuss treatment	1	**0**.**561**	0.439	—	—	—	—	—	—
	2	**0**.**857**	0.143	—	—	—	—	—	—
	3	0.288	**0**.**420**	0.293	—	—	—	—	—
Appointments per month	1	0.099	**0**.**617**	0.285	—	—	—	—	—
	2	**1**.**000**	—	—	—	—	—	—	—
	3	—	**0**.**592**	0.408	—	—	—	—	—
Financial impact	1	0.316	**0**.**684**	—	—	—	—	—	—
	2	**0**.**524**	0.286	0.190	—	—	—	—	—
	3	0.413	**0**.**476**	0.112	—	—	—	—	—

In Table 3, the “conditional probability” columns reflect the probability that an individual from a latent class 1–3 gives a response from 1 to 8 to a survey question (item). Columns 1–8 are the coded values of responses to each survey question, as shown in Table 1. For example, for the item “What is the received therapy type,” there are eight possible answers, where 1 = Antibody Therapy, 2 = No started therapy, etc. Most questions have three to four possible answers; therefore, the conditional probabilities for values above five are not estimated.

**Figure 3:**
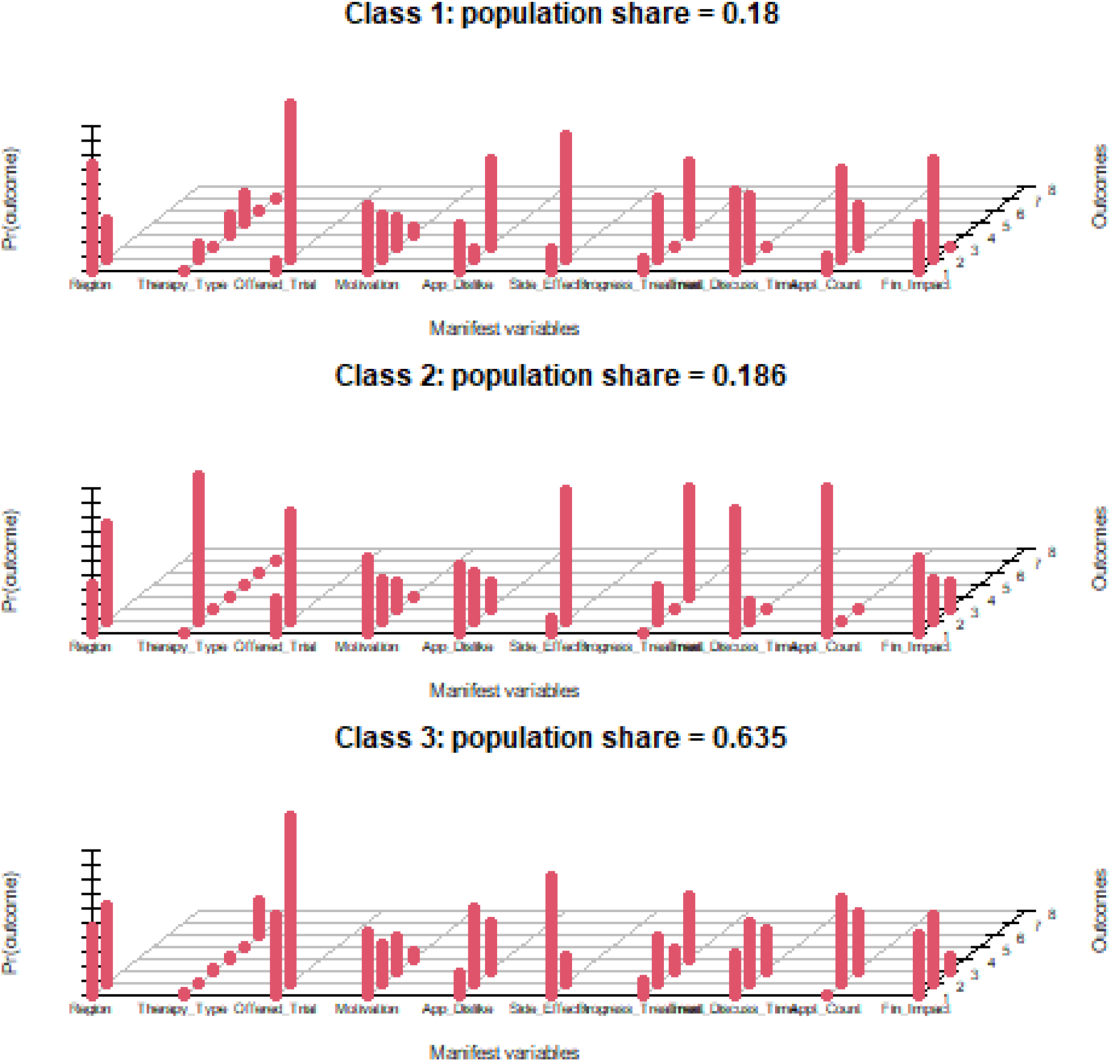
LCA distribution probability characteristics (source: SPSS).

The presented results outline the probabilities of a patient from a certain class endorsing an item. The highest conditional probability values for each variable and class are in bold. Figure 3 is a graphical representation of the conditional probability characteristics estimated by the fitted model.

Conditional probabilities help create a profile for each latent class by showing the likelihood of different responses within each class. Higher probabilities (taller red bars) indicate typical responses for that class, whereas lower probabilities indicate less typical responses.

Based on the patterns of high and low conditional probabilities, we have assigned descriptive labels to each latent class describing the most prominent traits observed within the class. The formation of these labels is derived from the most class-descriptive traits.

**Figure 4:**
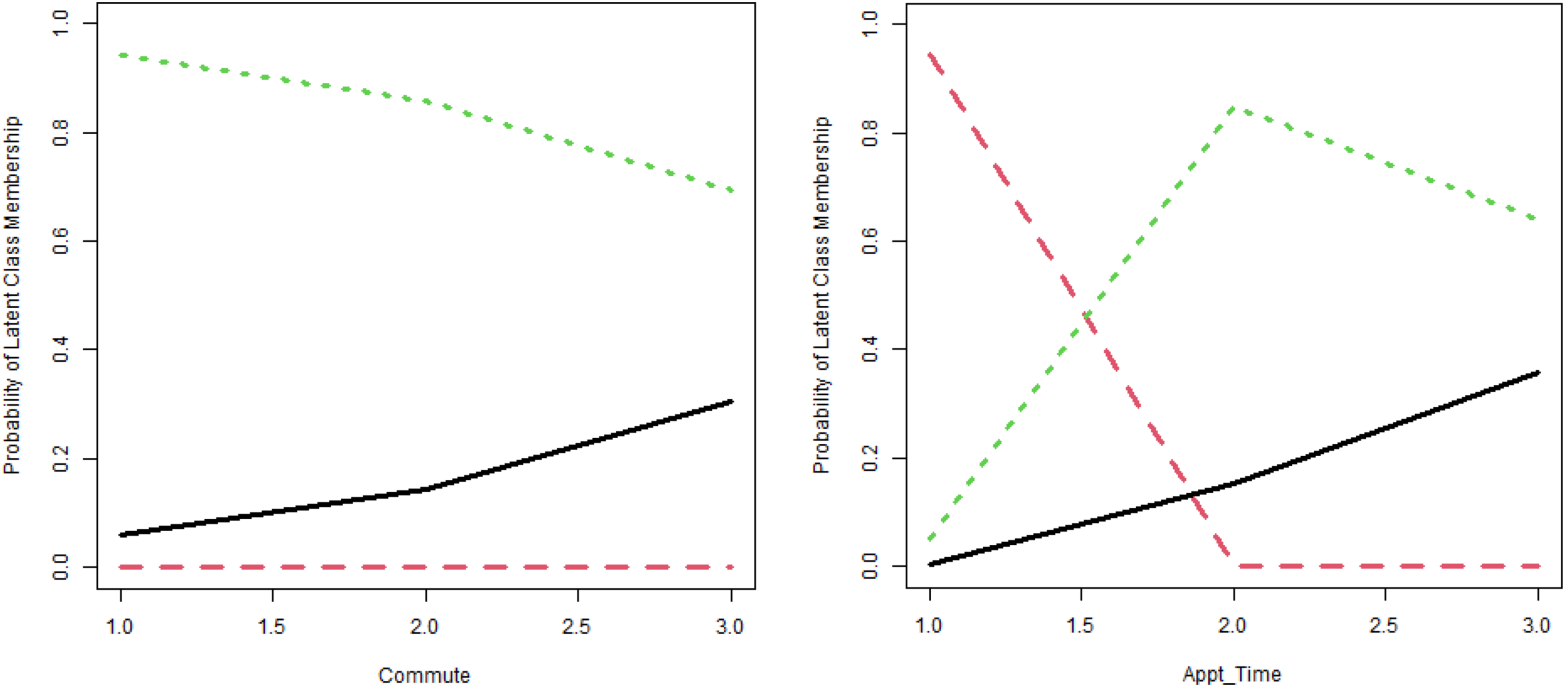
Probability of latent class membership (source: SPSS). Black line, LC 1; red line, LC 2; green line, LC 3.

Covariate regressions of the latent classes against the ordinal measurements for commute time (Commute) and average appointment duration (Appointment_Time) are presented in Figure 4.

The LCA model results provide insights into the underlying structure of the observed patient population across the three latent classes with distinct characteristics based on their responses to the measured variables. Figures in brackets show the conditional probabilities.

#### 4.1.1 Latent class 1: active treatment, moderate engagement

The results of the LCA for latent class 1 are presented in Table 4. For each row, heatmapping is used to illustrate the range of conditional probability values (darker colors indicate a higher probability). This class encompasses a moderate share of the patient population (18.0%), of which a predominant group is from Germany (73.2%).

**Table 4:** Estimated conditional probabilities for latent class 1.

Class I	1	2	3	4	5	6	7	8
Region	**0.732**	0.268	—	—	—	—	—	—
Therapy type	—	0.099	—	0.135	0.197	—	—	**0.569**
Offered trial	0.068	**0.932**	—	—	—	—	—	—
Motivation	**0.447**	0.302	0.201	0.050	—	—	—	—
Appointment dislike part	0.322	0.069	**0.610**	—	—	—	—	—
Side effects	0.154	**0.846**	—	—	—	—	—	—
Progress treatment	0.086	**0.417**	—	0.497	—	—	—	—
Time to discuss treatment	0.561	**0.439**	—	—	—	—	—	—
Appointments per month	0.099	**0.617**	0.285	—	—	—	—	—
Financial impact	0.316	**0.684**	—	—	—	—	—	—

#### 4.1.2 Latent class 2: early diagnostics, low engagement

The results of the LCA for latent class 2 are presented in Table 5. For each row, heatmapping is used to illustrate the range of conditional probability values (darker colors indicate a higher probability). This class encompasses a moderate share of the patient population (18.6%), predominantly from the USA (66.7%).

**Table 5:** Estimated conditional probabilities for latent class 2.

Class II	1	2	3	4	5	6	7	8
Region	**0.333**	0.667	—	—	—	—	—	—
Therapy type	1.000	—	—	—	—	—	—	
Offered trial	0.238	**0.762**	—	—	—	—	—	—
Motivation	**0.524**	0.286	0.190	—	—	—	—	—
Appointment dislike part	0.476	0.333	**0.190**	—	—	—	—	—
Side effects	—	**1.000**	—	—	—	—	—	—
Progress treatment	—	—	0.274	0.726	—	—	—	—
Time to discuss treatment	0.857	**0.143**	—	—	—	—	—	—
Appointments per month	1.000	—	—	—	—	—	—	—
Financial impact	0.524	**0.286**	0.190	—	—	—	—	—

#### 4.1.3 Latent class 3: active treatment, high engagement

The results of the LCA for latent class 3 are presented in Table 6. For each row, heatmapping is used to illustrate the range of conditional probability values (darker colors indicate a higher probability). This class encompasses a major share of the patient population (63.5%), balanced between Germany and the USA.

**Table 6:** Estimated conditional probabilities for latent class 3.

Class III	1	2	3	4	5	6	7	8
Region	**0.462**	0.538	—	—	—	—	—	—
Therapy type	0.028	—	0.014	0.018	—	0.237	0.056	**0.648**
Offered trial	0.525	**0.475**	—	—	—	—	—	—
Motivation	**0.445**	0.263	0.236	0.056	—	—	—	—
Appointment dislike part	0.146	0.524	**0.330**	—	—	—	—	—
Side effects	0.821	**0.179**	—	—	—	—	—	—
Progress treatment	0.101	**0.314**	0.153	0.431	—	—	—	—
Time to discuss treatment	0.288	**0.420**	0.293	—	—	—	—	—
Appointments per month	—	**0.592**	0.408	—	—	—	—	—
Financial impact	0.413	**0.476**	0.112	—	—	—	—	—

### 4.2 Hybrid IRT model

The rationale behind employing a hybrid IRT model is rooted in its ability to address the complexity of patient survey data. By combining different IRT models, the analysis can capture unidimensional and multidimensional aspects of the examined latent traits. Table 7 illustrates the summary results of the hybrid IRT model. Supplementary Material S2 presents the complete output.

**Table 7:** Hybrid IRT models results—summary.

(A) 2PL analysis		
Rcvd_Therapy	Discrim (a)	Diff (b)
Coef.	33.7215	0.7392
*P* > z	0.1730	0.0000
Offered_Trial		
Coef.	0.7276	0.7399
*P* > z	0.0090	0.0390
Side_Effects		
Coef.	1.5871	−0.1324
*P* > z	0.0000	0.4330
Off_Treatment		
Coef.	12.2257	0.9345
*P* > z	0.0000	0.0000
Disease_Prog		
Coef.	0.8514	0.1351
*P* > z	0.0020	0.5880

(B) GPCM analysis				
Time_Physi∼n	Discrim (1)	Diff (b)	2 vs. 1	3 vs. 2
Coef.	0.7807		0.1684	1.3527
*P* > z	0.0000		0.5750	0.0010
Appt_Time			2 vs. 1	3 vs. 2
Coef.	1.8165		−1.0918	1.2037
*P* > z	0.0000		0.0000	0.0000
Appt_Count			2 vs. 1	3 vs. 2
Coef.	2.7131		−0.8843	0.6631
*P* > z	0.0000		0.0000	0.0000
Commute			2 vs. 1	3 vs. 2
Coef.	1.0594		−2.5189	1.8438
*P* > z	0.0010		0.0000	0.0000
Fin_Impact			2 vs. 1	3 vs. 2
Coef.	0.1688		−0.8205	9.0649
*P* > z	0.3000		0.5620	0.2970

(C) NRM analysis								
Region	Discrim (a)	2 vs. 1	Diff (b)	2 vs. 1				
Coef.		−0.2315		0.2646				
*P* > z		0.2850		0.7550				
Therapy_Type	Discrim (a)	2 vs. 1	3 vs. 1	4 vs. 1	5 vs. 1	6 vs. 1	7 vs. 1	8 vs. 1
Coef.		−35.6284	−6.4819	−2.7591	−2.6149	−0.5311	−4.2640	−1.6578
*P* > z		0.1480	0.5680	0.1620	0.2060	0.7260	0.1210	0.2740
Therapy_Type	Diff (b)	2 vs. 1	3 vs. 1	4 vs. 1	5 vs. 1	6 vs. 1	7 vs. 1	8 vs. 1
Coef.		−0.5544	−0.0059	0.7613	0.7955	4.8890	0.4789	2.7006
*P* > z		0.0000	0.9940	0.0900	0.1080	0.6710	0.2660	0.1230
Motivation	Discrim (*a*)	2 vs. 1	3 vs. 1	4 vs. 1	Diff (b)	2 vs. 1	3 vs. 1	4 vs. 1
Coef.		−0.0622	0.0448	0.4384		−8.3240	16.3997	5.5522
*P* > z		0.7990	0.8670	0.5240		0.8000	0.8670	0.4990
App_Dislike	Discrim (a)	2 vs. 1	3 vs. 1	Diff (b)	2 vs. 1	3 vs. 1		
Coef.		0.8433	0.7648		−0.7604	−0.6778		
*P* > z		0.0040	0.0100		0.0470	0.0950		
Off_Treat_∼s	Discrim (a)	2 vs. 1	3 vs. 1	Diff (b)	2 vs. 1	3 vs. 1		
Coef.		14.5399	15.5584		1.1990	1.2610		
*P* > z		0.0000	0.0990		0.0000	0.0000		
Progress_T∼t	Discrim (a)	2 vs. 1	3 vs. 1	4 vs. 1	Diff (b)	2 vs. 1	3 vs. 1	4 vs. 1
Coef.		−1.1737	−0.2381	−1.7816		1.7037	1.5969	1.3659
*P* > z		0.1180	0.7770	0.0200		0.0230	0.6570	0.0010

## 5 Discussion

The interpretation of the results presented in Section 4 will be discussed within the latent classes identified in the LCA model and within the latent traits observed for the IRT model.

### 5.1 LCA

#### 5.1.1 Latent class 1—active treatment, moderate engagement

• Disease impact on daily life—patients under active treatment, mainly chemotherapy + (56.9%) but also radiotherapy and surgery. It is highly likely (84.6%) that patients in this group will not report side effects from their treatment. However, from a medical perspective, these types of treatment almost always lead to some degree of side effects. Most likely, this patient group experienced side effects but did not mention them. In addition, nearly half have disease progression with standard of care (SoC) treatment (49.7%). Two-thirds of the patients in this group are likely to report that the disease causes a moderate financial impact on their lifestyle (68.4%). Also, most members of this latent class (61%) are likely to be dissatisfied with organizational-related issues.
• Healthcare engagement—patients in this class spend low to moderate amounts of time discussing treatment with their physician. Patients in this group have not been offered the chance to join a clinical trial (93.2%) or receive an alternative therapy. They are motivated to join a trial for survival (44.7%) but also to find a cure and improve the quality of their life.
• Logistical challenges—most members of this group make moderate numbers of visits (2–5) per month, with an average appointment time between 60 and 120 min and are likely to report a moderate commute time.

Patient-centric suggestions based on the observed patient feasibility latent traits:

This latent class represents mainly German patients who can benefit from improved organizational treatment aspects such as more optimized visit schedules, a reduced number of appointments per month, and the provision of some form of travel assistance.

Individuals in this class seek to join clinical trials to improve their chances for survival, but their physicians seem to have a very limited awareness of such options. An improved clinical trial design targeting this group would need a robust outreach program, an information campaign, and support from patient organizations to improve awareness. Providing additional educational materials and counseling to patients with disease progression can improve their emotional experiences and reduce anxiety.

The observations presented in this class are consistent with previous studies (19) that highlighted the importance and impact of palliative care and especially the efficacy of care delivery in glioblastoma patients with progression. Furthermore, Preusser et al. focused on the impact that a tailored strategy for symptom and complication management can have on the perceived quality of life of these patients (20).

#### 5.1.2 Latent class 2—early diagnostics, low engagement

• Disease impact on daily life—this group exclusively represents patients whose active therapy has not started yet; therefore, they have not experienced any side effects yet. However, a small number of these individuals (27.6%) report evidence of disease progression. Most patients experience no financial impact on their lifestyle yet. A third of the patient population in latent class 2 are dissatisfied with the diagnostic process.
• Healthcare engagement—the majority of patients (85.7%) in this pre-treatment phase reported very low healthcare engagement with their physician and a lack of sufficient information. A very low proportion (23.8%) of the patients in latent class 2 are offered the chance to join a clinical trial but they actively seek participation to improve their chances of survival (52.4%), find a cure (28.6%), and improve their quality of life (19%).
• Logistical challenges—limited, as the active treatment has not started yet.

Patient-centric suggestions based on the observed patient feasibility latent traits:

Clinical trial design for patients in the early diagnostics phase should focus on creating more meaningful and helpful interactions between healthcare providers and patients. This can include extended consultation times and more detailed treatment discussions. Similar to the previous latent class, there is very limited awareness among these patients about the opportunities for joining a clinical trial, which can be improved through pre-trial advertising campaigns and support from advocacy groups.

Although there have been few studies exploring the perspectives of newly diagnosed glioblastoma patients, the observations pertaining to latent class 2 are consistent with the findings of authors such as Fritz et al. (21), who pinpointed the role of care planning, especially in the newly diagnosed patient setting. Furthermore, our findings in this class are in concert with the role of speed in starting glioblastoma treatment and the underlying patient motivation of improving survival chances underlined by Sun et al. (22).

#### 5.1.3 Latent class 3—active treatment, high engagement

• Disease impact on daily life—patients under active treatment, mainly chemotherapy + (56.9%) and surgery (23.7%). Very high probability of experiencing side effects (82.1%). Prevalence of people without disease progression (43.1%) and patients with disease progression under SoC treatment (31.4%). Notably, 15.3% of the individuals experience disease progression but have not been offered a treatment. The majority of these patients are dissatisfied with treatment-related aspects. The financial impact is none to medium, although some members report a severe financial burden (11.2%).
• Healthcare engagement—patients in this class exhibit the highest levels of engagement compared with the other two classes. They report longer times discussing treatment with their physician, and more than half have been offered the chance to join a clinical trial (52.5%). Their main motivation is to improve their survival chances (44.5%) and find a cure, but place a slightly higher probability on improving the quality of life, compared with the other classes.
• Logistical challenges—this patient class is associated with the highest frequency of monthly visits with their physician, longer commute times, and longer appointment durations.

Patient-centric suggestions based on observed patient feasibility latent traits:

This class represents the majority of patients under active therapy. Most of them experience side effects and report dissatisfaction with treatment-related aspects, including a slower recovery, pain or discomfort, and a deterioration in sleep quality. Clinical trial design can be improved to address these side effects by providing robust patient assistance, personalized support services, and easier access to specialists. Implementing a monitoring system to identify further side effects and disease progression early can improve the patient experience and minimize the burden on mental health. Although these group members have a higher probability of joining a clinical trial than the others, the clinical trial design still needs to increase awareness significantly.

The logistical challenges can be improved through more flexible visit schedules to minimize discomfort, especially for patients experiencing side effects. Implementing technologies for remote monitoring and video conferencing might also reduce the need for patients to travel for certain evaluations.

Contrary to the initial expectation, “motivation” follows the same probability distribution across all latent classes, suggesting that it has limited informative power on improving patient feasibility compared with the other observed variables. To explore what the most informative variables are and how they discriminate and relate to individual patients, the study employs the NRM from the IRT framework.

The findings and interpretations of latent class 3 show a group of people with high scores throughout all three latent traits, indicating a developed experience within the glioblastoma treatment. Patients in this group are generally dissatisfied with the treatment objectives and are actively seeking alternative options. These findings are consistent with previous studies highlighting patterns and disparities of care in glioblastoma, especially the influence of patient motivation (23). Furthermore, pertaining to the high score of healthcare engagement present in this class, Musella et al. (24) further defined the crucial role of shared decision-making for glioblastoma patients.

### 5.2 Hybrid IRT

#### 5.2.1 Disease impact on daily life

• **Region**: Difficulty and discrimination parameters are statistically insignificant; therefore, this item is not informative about the different levels of *disease impact*.
• **Received therapy**: The discrimination parameter is notably high but lacks statistical significance (*p* = 0.173). However, a substantially positive difficulty parameter of 0.73919 that is highly significant indicates that individuals who experience a higher *disease impact* are more likely to have received therapy.
• **Therapy type**: All discrimination parameters for the different therapy types are statistically insignificant. This suggests the response to this question does not effectively differentiate patients with regards to different levels of *disease impact* on their life, as the majority of them are treated with different forms of standard of care.

Despite the lack of discrimination, two therapy types (2 and 4) are statistically significant, meaning the item is still relevant for assessing the specific characteristics that affect disease impact for patients under different treatments. In practical terms, this signifies that the question can be worded better or the categorization should be revised.

• **Side effects and disease progression**: The discrimination parameter for both questions is relatively high and statistically significant, implying that these items effectively distinguish between individuals based on the level of *disease impact* on their life. The difficulty parameters lack statistical significance, suggesting that patients easily discern whether the presence of side effects or disease progression impacts their daily life.
• **Disease progression treatment**: The negative statistically significant discrimination coefficient for level 4 (no disease progression) indicates that the item effectively shows that the lack of disease progression implies a lower *disease impact* on a patient’s life. On the other hand, the statistically significant difficulty parameters suggests that patients with different forms of disease progression have varying levels of perceived disease impact on their life.
• **Appointment dislike part**: Discrimination and difficulty parameters are significant between levels 2 and 3 compared with level 1. These variations indicate that this question is highly relevant and meaningful for capturing the perceived impact of the disease on the patient’s lifestyle.
• **Taken off treatment**: This item demonstrates a high discrimination parameter with statistical significance. The substantially positive difficulty parameter (0.934464, z = 8.34) indicates a robust connection between higher levels of perceived *disease impact* and a significantly increased likelihood of being taken off treatment due to side effects.
• **Off-treatment options**: There is substantial discrimination between levels 2 and 3 compared with level 1, supported by statistically significant difficulty parameters. These distinctions imply that the question effectively discriminates between patients under different off-treatment options. In addition, the item is informative about how different off-treatment options affect the perceived level of the latent trait (*disease impact*).
• **Financial impact**: Displays a statistically insignificant discriminative ability, suggesting limited effectiveness in differentiating individuals based on the latent trait. The difficulty parameters also lack statistical significance, indicating uncertainty regarding the association between different levels of the disease impact and financial impact.

Examining disease impact as a latent trait shows that the questions regarding the presence of side effects, disease progression, the availability of treatment options for patients with side effects, and disease progression, as well as the reported dissatisfaction with various treatment aspects, can provide valuable insights into how to improve patient feasibility.

**Figure 5:**
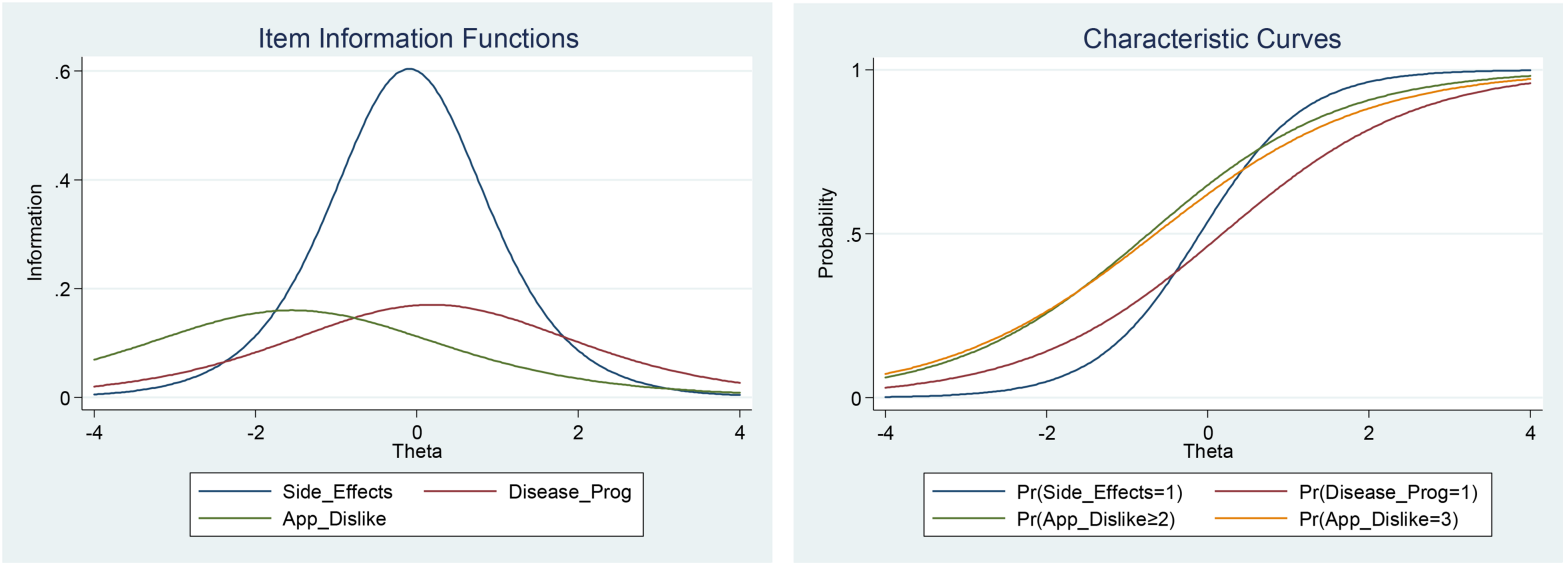
Item information function curves and item characteristic curves of selected items in the disease impact on daily life trait.

The item “received therapy type” also indicates a relevance to the level of disease impact; however, the question format or the data categorization must be revisited. On the other hand, the patient's country of origin and the financial impact do not seem to capture the important aspects associated with the perceived level of the latent trait (Figure 5).

#### 5.2.2 Healthcare engagement

• **Time for discussing treatment**: Displays moderate discrimination, suggesting the effectiveness of this question in discerning individuals based on their levels of *healthcare engagement*. The relatively high and statistically significant difficulty parameter for comparison between levels 3 and 1 (1.3527) provides evidence that patients who spend more time discussing treatment options with their physicians have higher *healthcare engagement*.
• **Offered the chance to join a clinical trial**: The discrimination parameter is moderate and statistically significant (*p* = 0.009), suggesting there is an association between higher *healthcare engagement* and an increased likelihood of being offered a trial. The positive difficulty parameter (0.739933) reinforces this relationship.
• **Motivation**: No clear discriminatory patterns are observed across different levels of motivation regarding the limiting factors in motivation and treatment. This result lends statistical support to the finding in the latent class analysis that the variable motivation is not informative as a measurement for patient feasibility within the current study.

**Figure 6:**
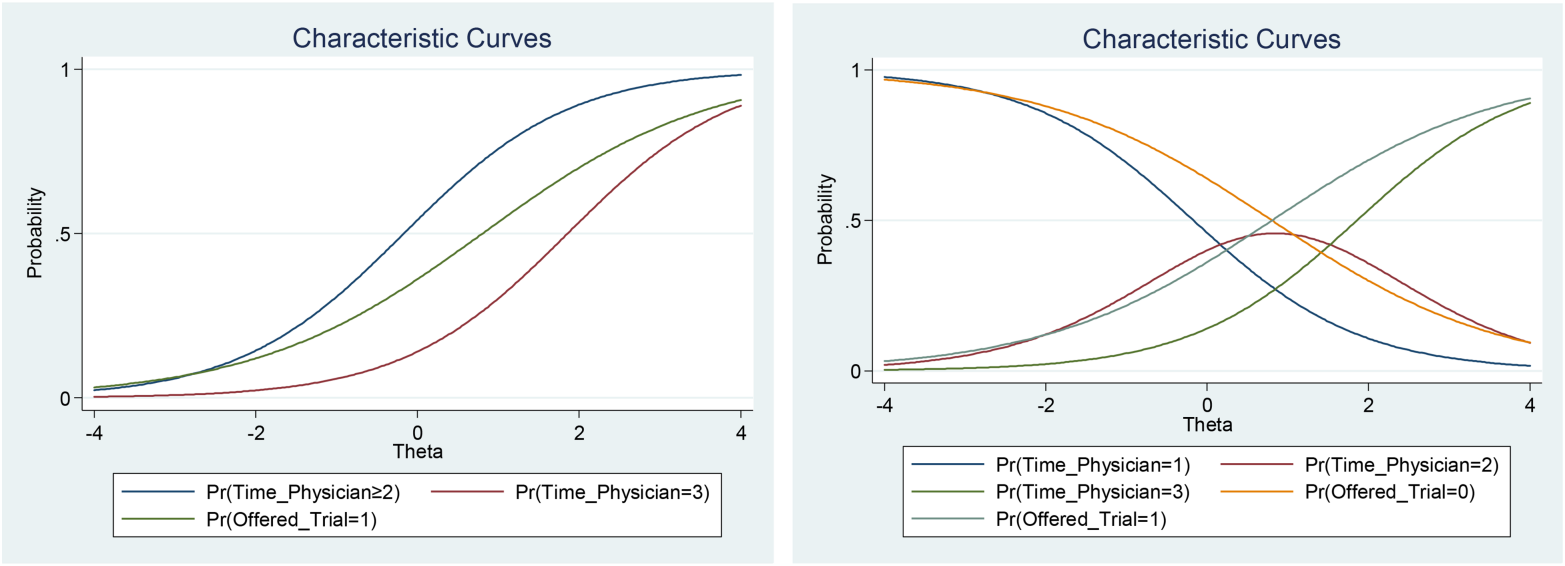
Item characteristic curves of selected items in the healthcare engagement trait.

The data seem to support the notion that longer patient–physician interactions lead to higher healthcare engagement. Spending more time with their physicians to discuss treatment allow patients to gain a better understanding of their treatment options and risks, allowing for more informed decision-making. It also builds trust, improving the adherence to prescribed treatment plans (Figure 6).

A patient's motivation to join a trial does not seem to affect their levels of engagement. However, data suggest that the opportunity to join a trial is of significant importance. Access to trials can contribute to higher levels of engagement because patients seek to improve their survival chances with novel treatment options and benefit from contact with highly experienced healthcare professionals, regardless of their motivation (Figure 6).

#### 5.2.3 Logistic challenges

##### 5.2.3.1 Appointment duration

**Figure 7:**
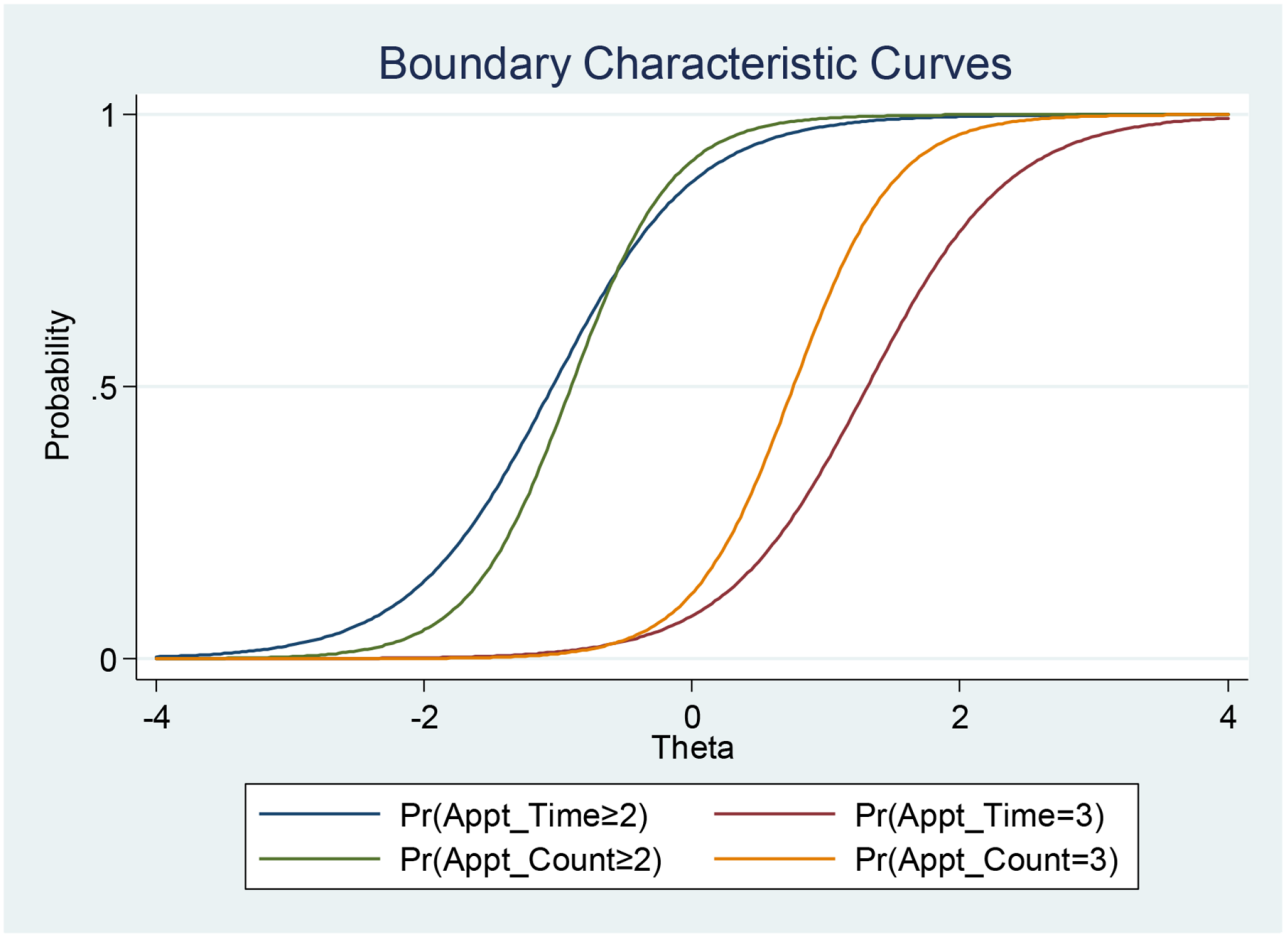
Boundary characteristic curves for selected items in the logistic challenges trait.

Appointment duration exhibits a substantial discriminative ability with high significance (1.8165), effectively differentiating individuals based on the perceived logistical challenges. The negative difficulty parameters for comparisons 2 vs. 1 and positive parameters for 3 vs. 2 suggest that a medium appointment duration is not perceived as a meaningful *logistic challenge*, but further increases in the appointment time are associated with a higher patient burden (Figure 7).

##### 5.2.3.2 Appointments per month

Results showed that appointments per month has a high discriminative ability, indicating the effectiveness of this question in differentiating individuals based on perceived logistical challenges. There are similar changes in the latent trait across appointment count levels, supported by the negative difficulty parameter for comparison 2 vs. 1 and the positive parameter for 3 vs. 2.

##### 5.2.3.3 Commute

Commute duration demonstrates moderate discrimination, effectively distinguishing individuals based on the perceived *logistic challenges*. The negative difficulty parameter for comparison 2 vs. 1 and positive parameter for 3 vs. 2 implies changes in the latent trait across varying commute durations (Figure 7).

The observed findings in the IRT analysis demonstrate a purely patient-derived picture of the perceived disease burden, experienced logistical challenges pertaining to treatment, and the quality of the engagement patients have with the healthcare system. These findings are conclusive, with other studies reporting a significant disparity between expert-reported and patient-reported disease rankings, such as the study by Broekharst et al. (25). The described findings in this section can potentially shed light on the quality of care for glioblastoma with standard of care treatment outside of the patient feasibility model proposed in this study. Such implications have also been observed by Banerji et al., in which the patient-reported burden of hereditary angioedema is presented as a flexible construct that seems to change after interventions such as prophylactic treatment (26).

### 5.3 Advantages and limitations

#### 5.3.1 Advantages

LCA and interval response theory are two statistical methods commonly used in patient-reported outcomes and patient surveys. LCA is a valuable tool for identifying distinct subgroups within a sample, which can aid in risk stratification and treatment response prediction (27).

Studies have successfully applied LCA to classify patients into different phenogroups based on outcomes after surgeries such as mitral valve surgery (27, 28). This approach allows for a more personalized understanding of patient responses and outcomes.

On the other hand, interval response theory, as demonstrated in previous studies (29), provides a method for capturing richer information by allowing respondents to provide interval-valued responses. This can be particularly useful in scenarios in which respondents may have difficulty providing precise single responses, as it allows for a range of values to be considered (29). By incorporating interval responses, more nuanced data can be obtained from patients.

Although not new, LCA and IRT statistical models have the power to use mixes of ordinal and nominal data, which are essential parts of data collection tools such as patient surveys. By harnessing the impact of IRT models, the importance and nuance within different patient-reported outcomes can be elaborated and the discriminating power of different data collection items (e.g., survey questions) can be evaluated.

In essence, while the IRT model provides a comprehensive overview of the item characteristics and latent traits observed, the latent classes from the LCA add a layer of contextual understanding that makes the surfaced insights actionable for a specific patient subgroup (30).

#### 5.3.2 Limitations

Although the previously mentioned models offer valuable advantages, it is also important to acknowledge their limitations. LCA and IRT models rely on certain assumptions, such as the unidimensionality and local independence of items, which may not always hold true in complex real-world scenarios (31).

In addition, the interpretation of latent classes and traits derived from these models requires careful consideration, as the results are based on statistical patterns and may not always align perfectly with clinical or practical significance. A mention in this context is the active treatment-moderate engagement class in this study. No side effects resulting from chemotherapy and radiotherapy were reported within the group; however, these patients most likely experienced some degree of side effects but probably did not mention them in the survey.

Furthermore, the generalizability of the identified latent classes and traits to broader patient populations should be approached with caution, as the specific characteristics of the sample under study may not be fully representative of other populations. Another limitation is the impracticality of applying statistical parameters from LCA models to individual new patients, which has been noted as a limitation in clinical situations (32). In addition, the use of IRT models may be limited by the number of cases and features used to derive clusters, potentially impacting the reproducibility and generalizability of the findings (33).

Another potential limitation of this study is the application of the IRT model to the whole dataset rather than within each latent class. In its current form, the IRT is applied to the whole dataset to streamline the modeling process due to the complexity of the patient survey responses. Although this approach provides broad insights into item characteristics and latent traits, it may overlook important subgroup-specific differences in item parameters that could lead to less precise insights. Future improvement in this regard would be the incorporation of Likert-type responses that would introduce a standardized measurement scale across all items (34). This would create a prerequisite for using more complex analysis by integrating IRT within each latent class (35).

### 5.4 Future applications

A potential future application of the combined use of IRT and LCA like the presented framework is the development of more advanced disease burden scores, especially when combined with ML mechanisms. An example of this would be the study by Teunissen et al. (36), who utilized computerized adaptive testing based on IRT for the patient evaluation measure in individuals undergoing cubital tunnel syndrome surgery. By harnessing IRT, the study reduced the patient burden while increasing the construct validity, highlighting the efficiency and accuracy of IRT in assessing disease burden in clinical settings.

## 6 Conclusion

Patient feasibility as a concept at the crossroad of LCA and IRT analysis represents a promising tool for investigating qualitative latent traits and patterns within cohorts of patient data. Through LCA, prominent latent classes are outlined, whereas IRT provides more granular insight into the nuance within the patient responses. These tools can create analytical frameworks that uncover not only the latent traits and patterns within patient data but also the behavioral drivers that shape them.

## Data Availability

The original contributions presented in the study are included in the article/Supplementary Material, further inquiries can be directed to the corresponding author.

## References

[1] Getz KA. Establishing return-on-investment expectations for patient-centric initiatives. Ther Innov Regul Sci. (2015) 49(5):745–9. 10.1177/2168479015579521 30227037

[2] Oehrlein EM Harris J Balch A Furlong P Hargis E Woolley M Improving access and quality of health care in the United States: shared goals among patient advocates. Patient. (2020) 14(5):687–90. 10.1007/s40271-020-00453-4 PMC8357644 33083996

[3] Lim SY Kivitz A McKinnell D Pierson ME O'Brien F. Simulating clinical trial visits yields patient insights into study design and recruitment. Patient Prefer Adherence. (2017) 11:1295–307. 10.2147/ppa.s137416 28814837 PMC5545635

[4] Kern-Goldberger AS Hessels A Saiman L Quittell LM. Understanding of safety monitoring in clinical trials by individuals with CF or their parents: a qualitative analysis. J Cyst Fibros. (2018) 17(6):736–41. 10.1016/j.jcf.2018.01.011 29550263

[5] Jones SJ Flewett M Flewett R Lee S Vick B Thompson M Clinical trial simulations in pulmonary fibrosis: patient-focused insights and adaptations. ERJ Open Res. (2023) 9(3):00602–2022. 10.1183/23120541.00602-2022 37260456 PMC10227627

[6] Lalanza S Peña C Bezos C Yamauchi N Taffner V Rodrigues KL Patient and healthcare professional insights of home- and remote-based clinical assessment: a qualitative study from Spain and Brazil to determine implications for clinical trials and current practice. Adv Ther. (2023) 40(4):1670–85. 10.1007/s12325-023-02441-0 36795221 PMC9933016

[7] Gloy V Speich B Griessbach A Heravi AT Schulz A Fabbro T Scoping review and characteristics of publicly available checklists for assessing clinical trial feasibility. BMC Med Res Methodol. (2022) 22:1. 10.1186/s12874-022-01617-6 35590285 PMC9118562

[8] Evans S Paraoan D Perlmutter J Raman SR Sheehan JJ Hallinan ZP. Real-world data for planning eligibility criteria and enhancing recruitment: recommendations from the clinical trials transformation initiative. Ther Innov Regul Sci. (2021) 55(3):545–52. 10.1007/s43441-020-00248-7 33393014 PMC8021522

[9] Claire R Gluud C Berlin I Coleman T Leonardi-Bee J. Using trial sequential analysis for estimating the sample sizes of further trials: example using smoking cessation intervention. BMC Med Res Methodol. (2020) 20:284. 10.1186/s12874-020-01169-7 33256626 PMC7702700

[10] Barnett H. The Patient Perspective of Quality Care: A Literature Review. Washington DC: The George Washington University Undergraduate Review (2019). p. 2.

[11] Beardon S Patel K Davies B Ward H. Informal carers’ perspectives on the delivery of acute hospital care for patients with dementia: a systematic review. BMC Geriatr. (2018) 18:1. 10.1186/s12877-018-0710-x 29370769 PMC5785800

[12] Kuny AV Althoff RR Copeland WE Bartels M Van Beijsterveldt CEM Baer J Separating the domains of oppositional behavior: comparing latent models of the Conners’ oppositional subscale. J Am Acad Child Adolesc Psychiatry. (2013) 52(2):172–83.e8. 10.1016/j.jaac.2012.10.005 23357444 PMC3558689

[13] Buatois S Retout S Frey N Ueckert S. Item response theory as an efficient tool to describe a heterogeneous clinical rating scale in *de novo* idiopathic Parkinson’s disease patients. Pharm Res. (2017) 34(10):2109–18. 10.1007/s11095-017-2216-1 28695401

[14] Wu L Ling W Burchett BM Blazer DG Yang C Pan JJ Use of item response theory and latent class analysis to link poly-substance use disorders with addiction severity, HIV risk, and quality of life among opioid-dependent patients in the clinical trials network. Drug Alcohol Depend. (2011) 118(2–3):186–93. 10.1016/j.drugalcdep.2011.03.018 21501933 PMC3170493

[15] Ueckert S Plan EL Ito K Karlsson MO Corrigan B Hooker AC. Improved utilization of ADAS-cog assessment data through item response theory based pharmacometric modeling. Pharm Res. (2014) 31(8):2152–65. 10.1007/s11095-014-1315-5 24595495 PMC4153970

[16] Lui L Tan A Ng M Chow P Tan D. The impact of COVID-19 on clinical trials in the Asia-pacific region and future implications. Res Sq. (2021). 10.21203/rs.3.rs-429561/v1

[17] Kee D Blank LJ Kummer B Mazumdar M Agarwal P. Latent class analysis of ehealth behaviors among adults with epilepsy. Epilepsia. (2022) 64(2):479–99. 10.1111/epi.17483 36484565

[18] Jayedi A Neyestanak MS Djafarian K Shab-Bidar S. Temporal patterns of energy intake identified by the latent class analysis in relation to prevalence of overweight and obesity in Iranian adults. Br J Nutr. (2023) 130(11):2002–12. 10.1017/s000711452300096x 37132327

[19] Hemminger L Pittman C Korones DN Serventi J Ladwig S Holloway RG Palliative and end-of-life care in glioblastoma: defining and measuring opportunities to improve care. Neurooncol Pract. (2016) 4(3):182–8. 10.1093/nop/npw022 31385987 PMC6655415

[20] Preusser M de Ribaupierre S Wöhrer A Erridge S Hegi ME Weller M Current concepts and management of glioblastoma. Ann Neurol. (2011) 70(1):9–21. 10.1002/ana.22425 21786296

[21] Fritz L Dirven L Reijneveld JC Koekkoek JAF Stiggelbout AM Pasman HRW Advance care planning in glioblastoma patients. Cancers (Basel). (2016) 8(11):102. 10.3390/cancers8110102 27834803 PMC5126762

[22] Sun MZ Oh T Ivan ME Clark AJ Safaee M Sayegh ET Survival impact of time to initiation of chemoradiotherapy after resection of newly diagnosed glioblastoma. J Neurosurg. (2015) 122(5):1144–50. 10.3171/2014.9.jns14193 25768833

[23] Dressler EV Liu M García C Dolecek TA Pittman T Huang B Patterns and disparities of care in glioblastoma. Neurooncol Pract. (2018) 6(1):37–46. 10.1093/nop/npy014 30740232 PMC6352755

[24] Musella A DeVitto R Anthony M Mydland DE. The importance of shared decision-making for patients with glioblastoma. Patient preference and adherence. Volume. (2021) 15:2009–16. 10.2147/ppa.s314792 34531651 PMC8439973

[25] Broekharst DSE Bloem S Groenland EA Van Raaij WF Van Agthoven M. Differences between expert reported and patient reported burden of disease rankings. Sci Rep. (2022) 12(1):895. 10.1038/s41598-021-04070-5 35042859 PMC8766519

[26] Banerji A Davis KH Brown TM Hollis K Hunter S Long J Patient-reported burden of hereditary angioedema: findings from a patient survey in the United States. Ann Allergy Asthma Immunol. (2020) 124(6):600–7. 10.1016/j.anai.2020.02.018 32169514

[27] Kwak S Lee S Lim J Yang S Choi H Hwang I Long-term outcomes in distinct phenogroups of patients with primary mitral regurgitation undergoing valve surgery. Heart. (2022) 109(4):305–13. 10.1136/heartjnl-2022-321305 PMC9887360 35882521

[28] Messika–Zeitoun D Chan V Burwash IG. Latent class analysis to predict outcomes after surgery for primary mitral regurgitation: a scientific validation of common sense. Heart. (2022) 109(4):253–5. 10.1136/heartjnl-2022-321555 36270783

[29] Ellerby Z Wagner C Broomell SB. Capturing richer information: on establishing the validity of an interval-valued survey response mode. Behav Res Methods. (2021) 54(3):1240–62. 10.3758/s13428-021-01635-0 34494219 PMC9170647

[30] López-Valenciano A Ruiz-Pérez I Ayala F Sánchez-Meca J Vera-Garcia FJ. Updated systematic review and meta-analysis on the role of isometric resistance training for resting blood pressure management in adults. J Hypertens. (2019) 37(7):1320–33. 10.1097/HJH.0000000000002022 30624369

[31] Reise S Rodriguez A. Item response theory and the measurement of psychiatric constructs: some empirical and conceptual issues and challenges. Psychol Med. (2016) 46(10):2025–39. 10.1017/s0033291716000520 27056796

[32] Kongsted A Hestbæk L. How can latent trajectories of back pain be translated into defined subgroups? BMC Musculoskelet Disord. (2017) 18:1. 10.1186/s12891-017-1644-8 28673341 PMC5496263

[33] Koutroulis I Velez T Wang T Yohannes S Galarraga JE Morales JA Pediatric sepsis phenotypes for enhanced therapeutics: an application of clustering to electronic health records. J Am Coll Emerg Physicians Open. (2022) 3:1. 10.1002/emp2.12660 35112102 PMC8790108

[34] Kobayashi L Sweeney L Cousins AC Bertsch K Gardiner F Boss RM Web survey and embedded intervention on emergency department personnel perceptions of role in patient experience. Eur J Emerg Med. (2012) 19(2):112–6. 10.1097/mej.0b013e3283484b9f 21659883

[35] Schröder A Wilde-Larsson B Ahlström G Lundqvist L. Psychometric properties of the instrument quality in psychiatric care and descriptions of quality of care among in-patients. Int J Health Care Qual Assur. (2010) 23(6):554–70. 10.1108/09526861011060924 20845822

[36] Teunissen JS Hovius SE Ulrich D Issa F Rodrigues J Harrison C. Computerized adaptive testing for the patient evaluation measure (PEM) in patients undergoing cubital tunnel syndrome surgery. J Hand Surg Eur Vol. (2023) 48(10):1042–7. 10.1177/17531934231164959 37066610 PMC10616996

