# Supplementary material for: Patient feasibility as a novel approach for integrating IRT and LCA statistical models into patient-centric qualitative data—a pilot study
Source: Front Digit Health. 2024 Oct 2;6:1378497. doi: 10.3389/fdgth.2024.1378497 (PMC11479982; doi:10.3389/fdgth.2024.1378497)
Supplement: Supplementary file 1 [file Table1.docx]

# **Appendix**

## **Appendix I – Measurement Frequencies**

| Region  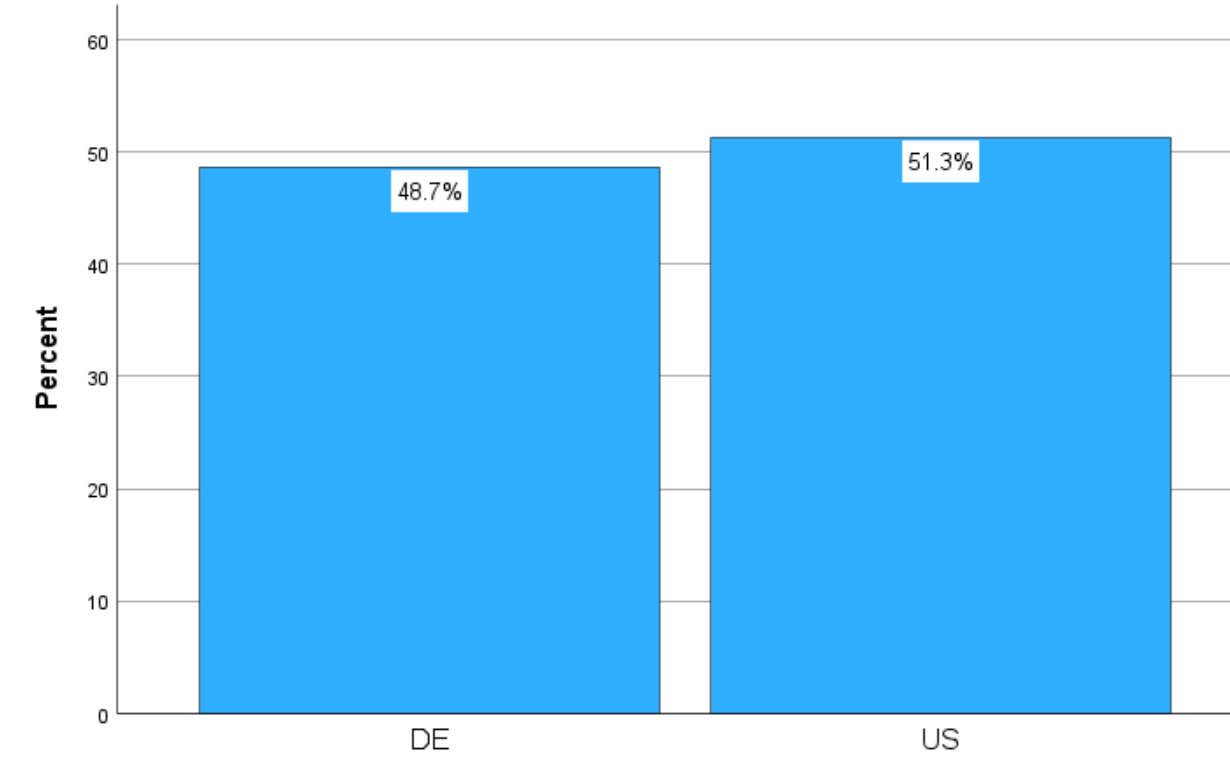 | Received Therapy  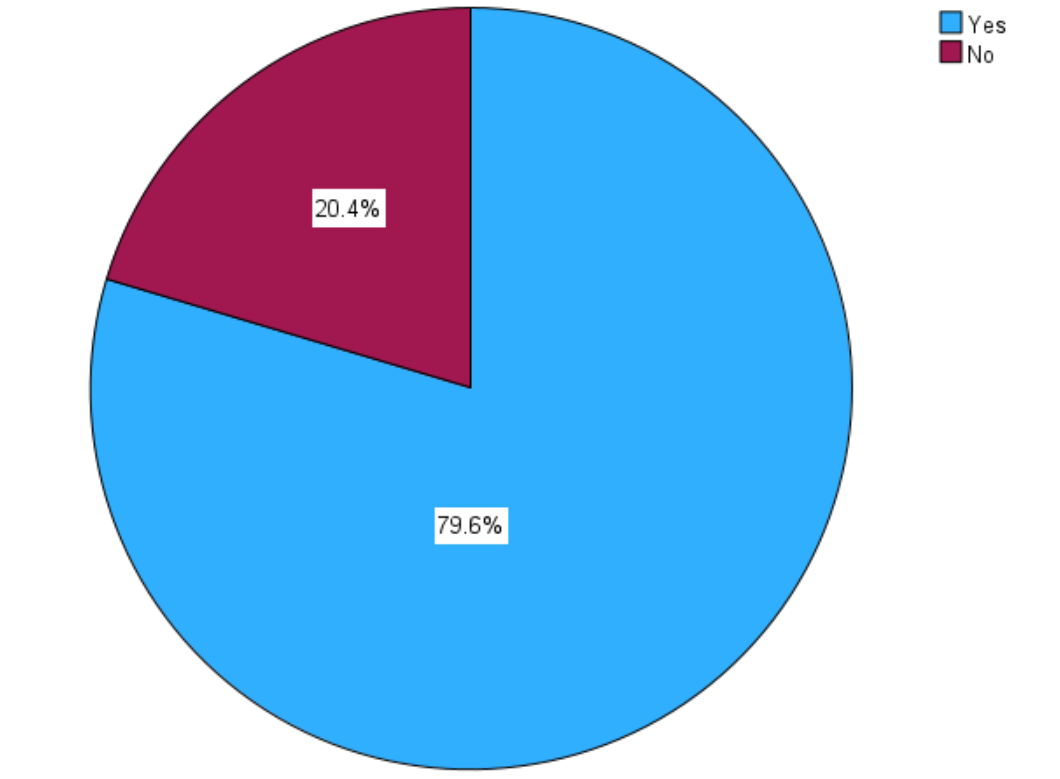 |
| --- | --- |
| Therapy Type  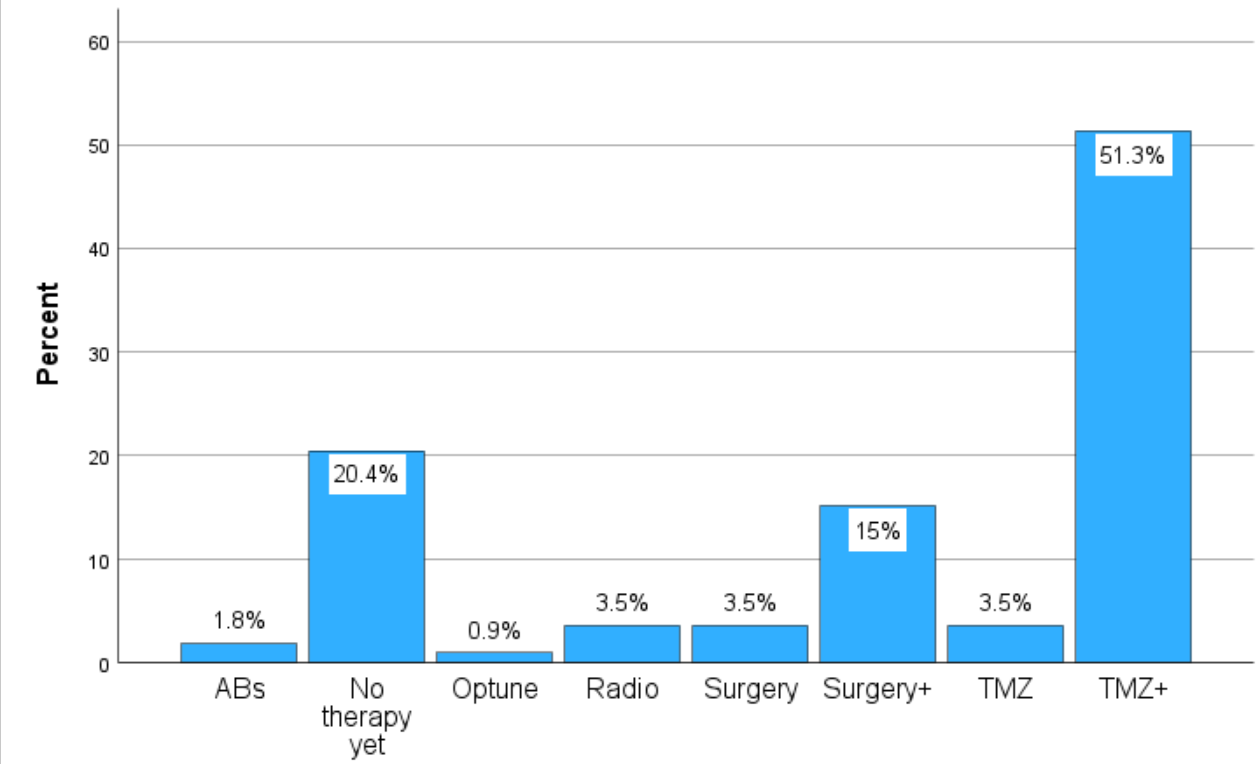 | Motivation  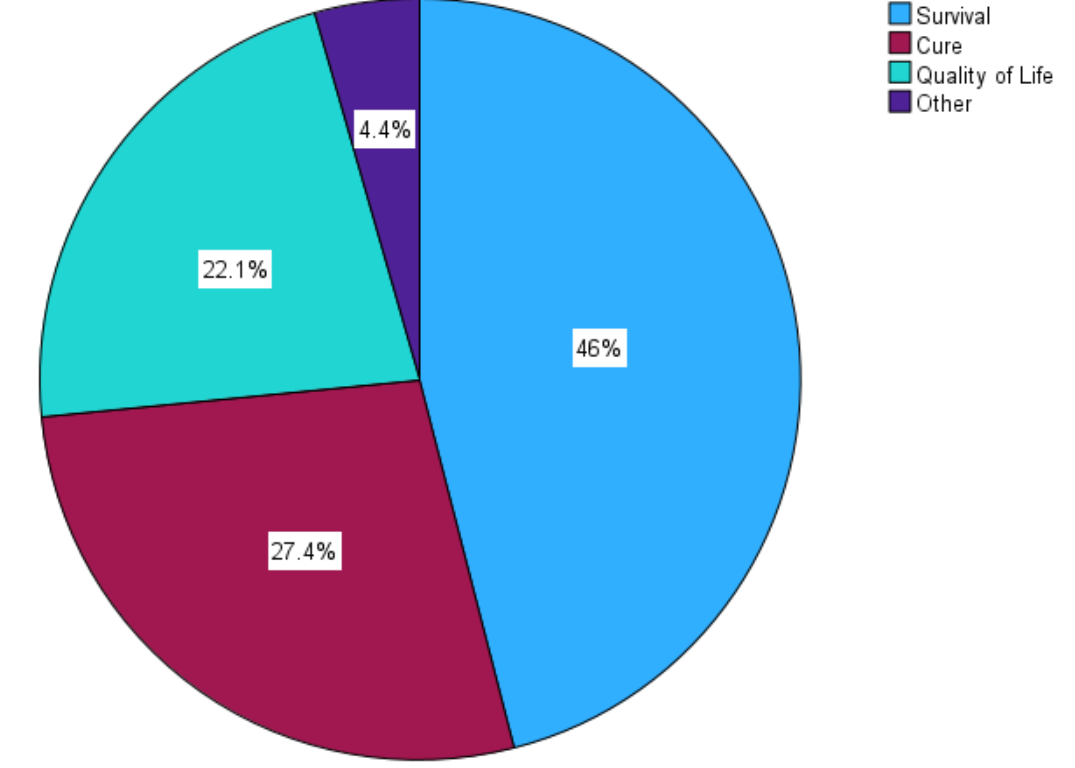 |
| Time to discuss treatment  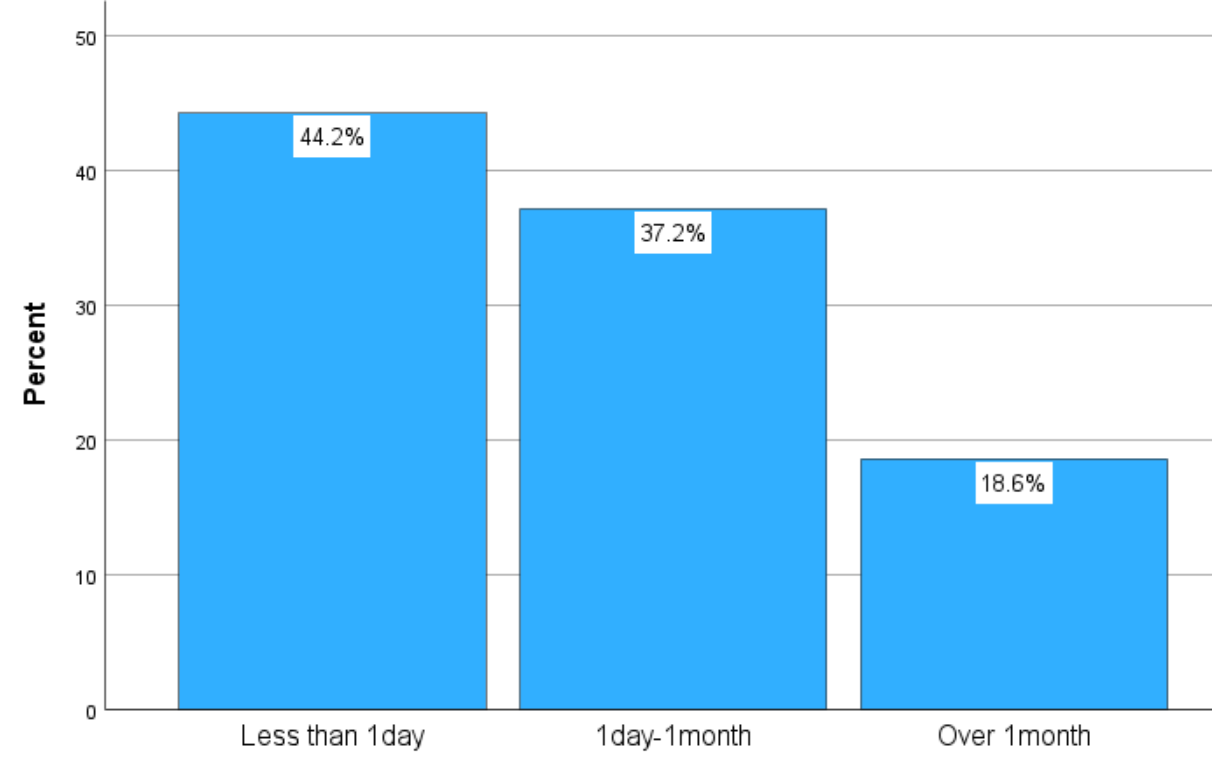 | Appointments per Month  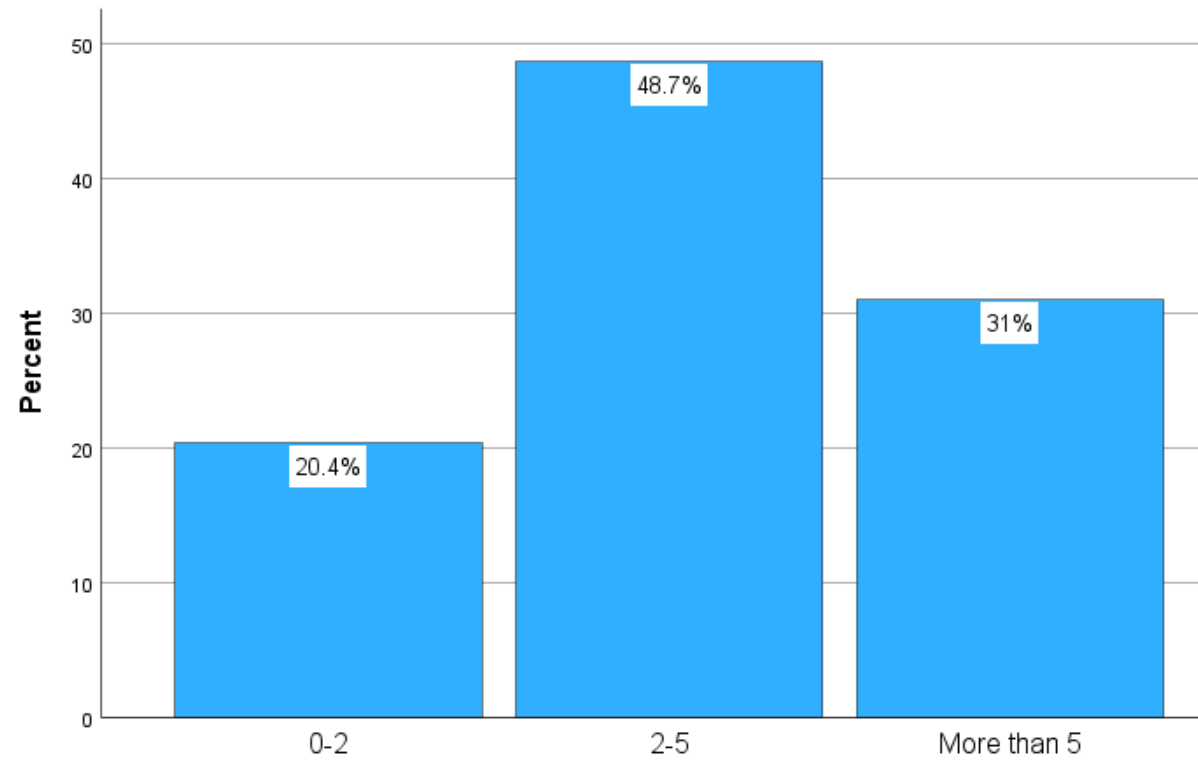 |
| Commute Time  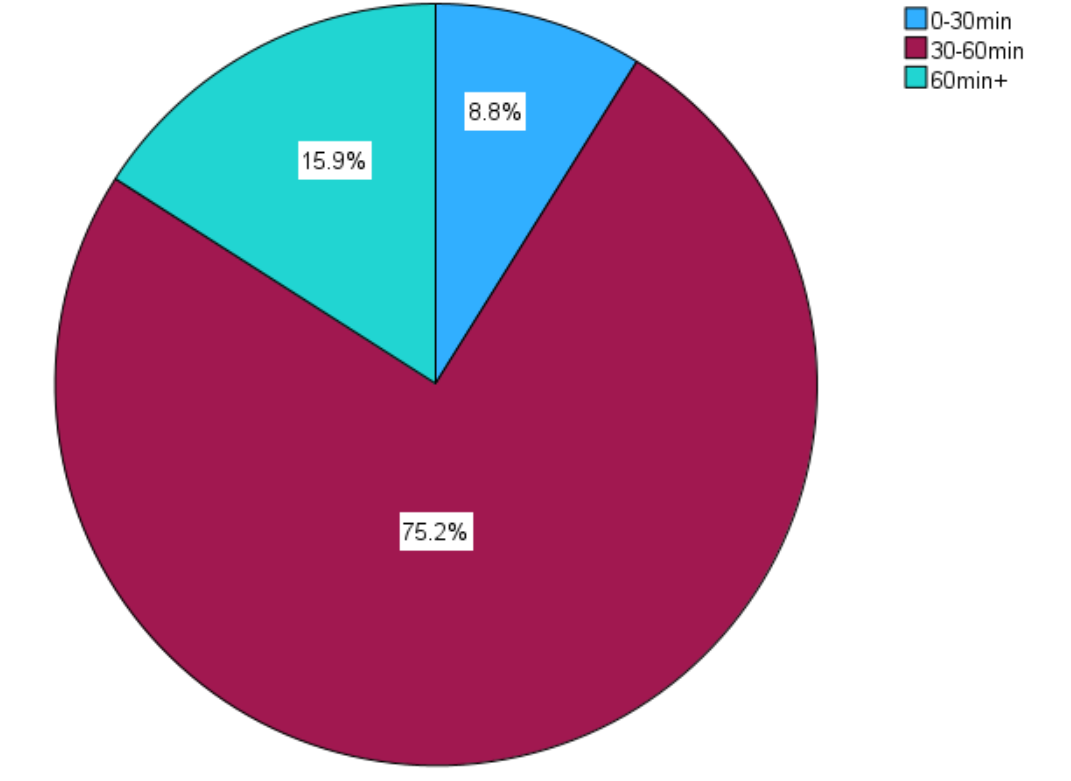 | Appointment Time  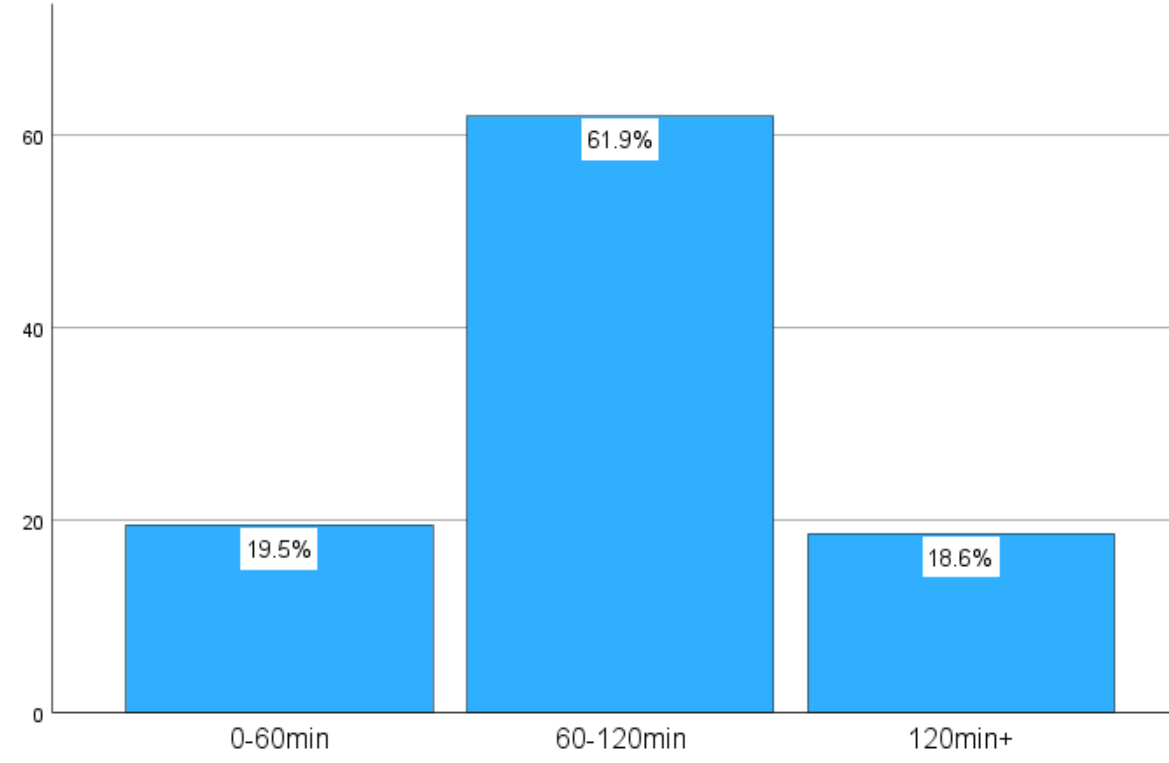 |
| Appointment Dislike Part  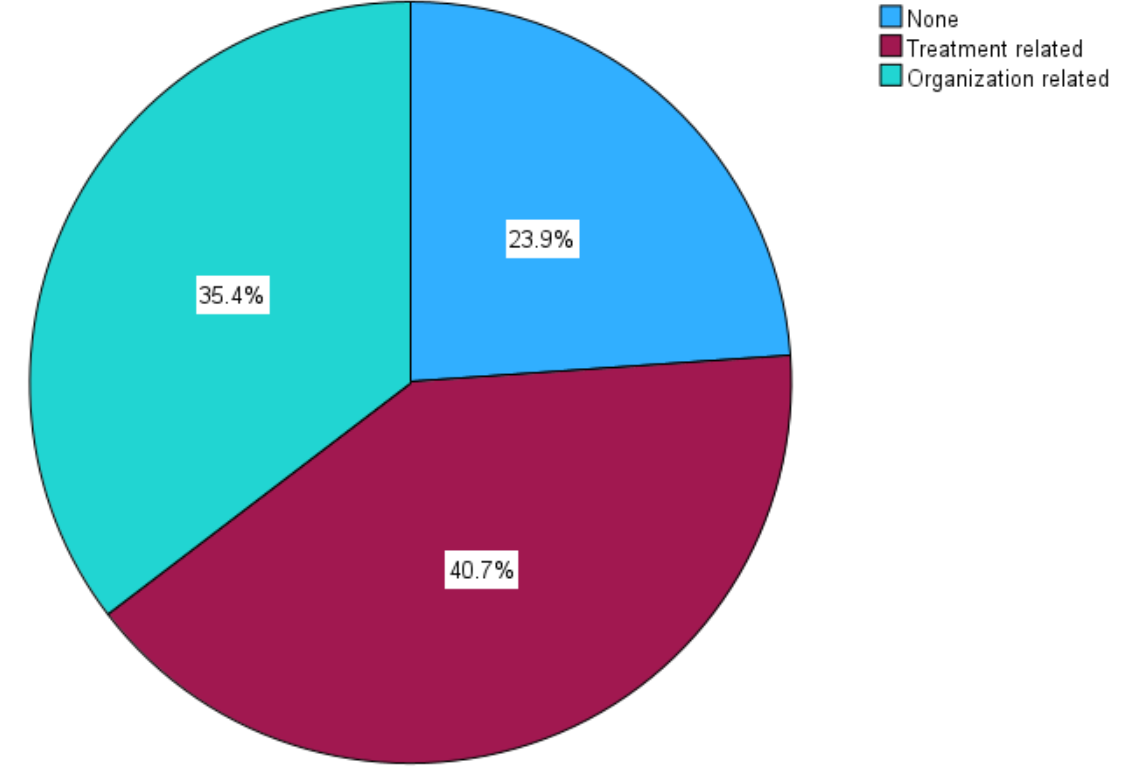 | Clinical Trial Offered  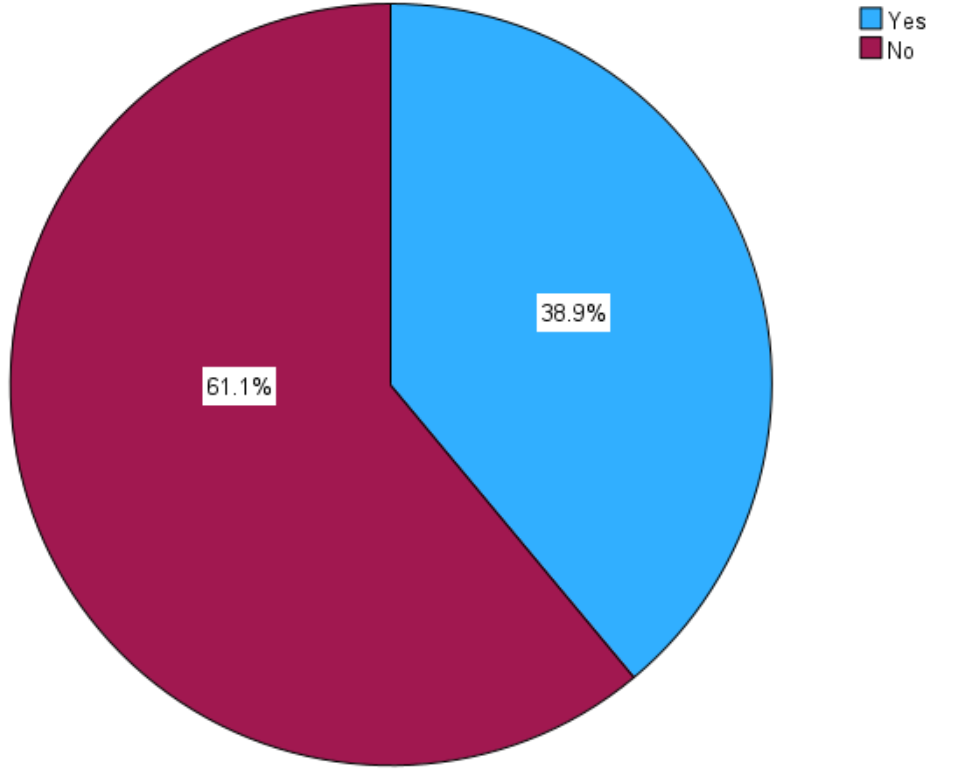 |
| Financial Impact  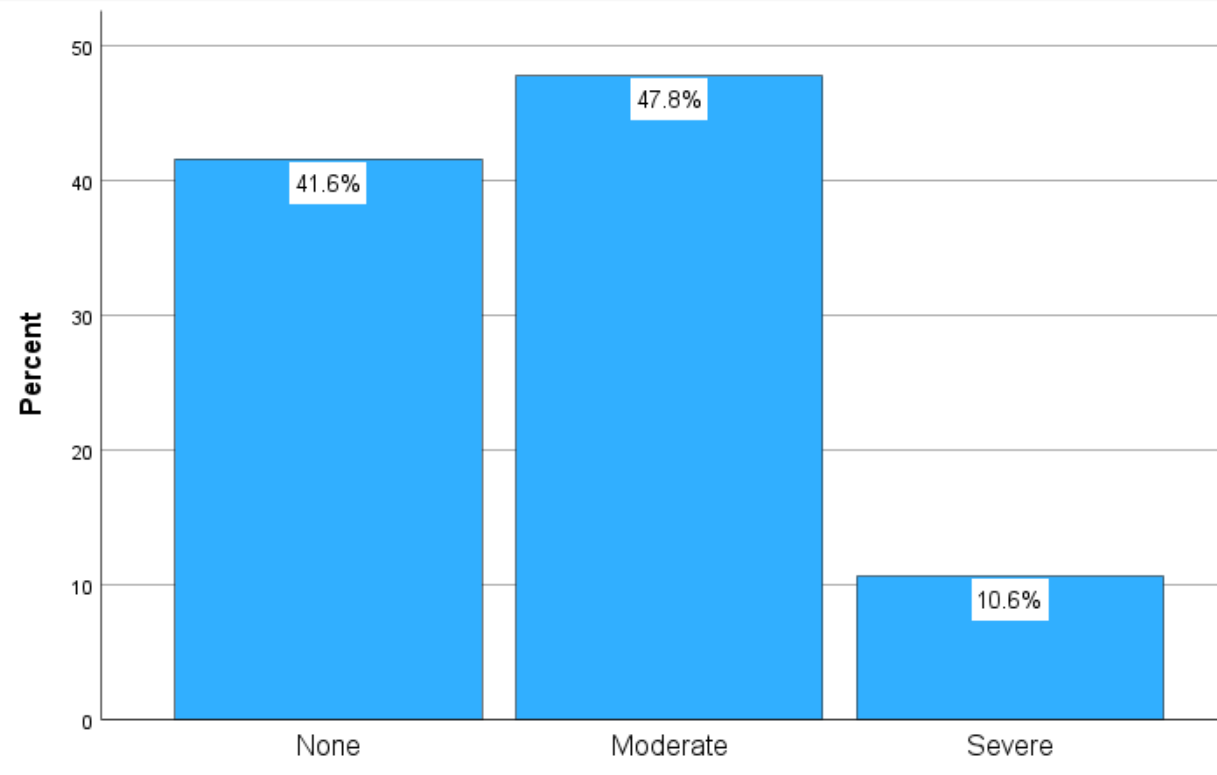 | Side Effects  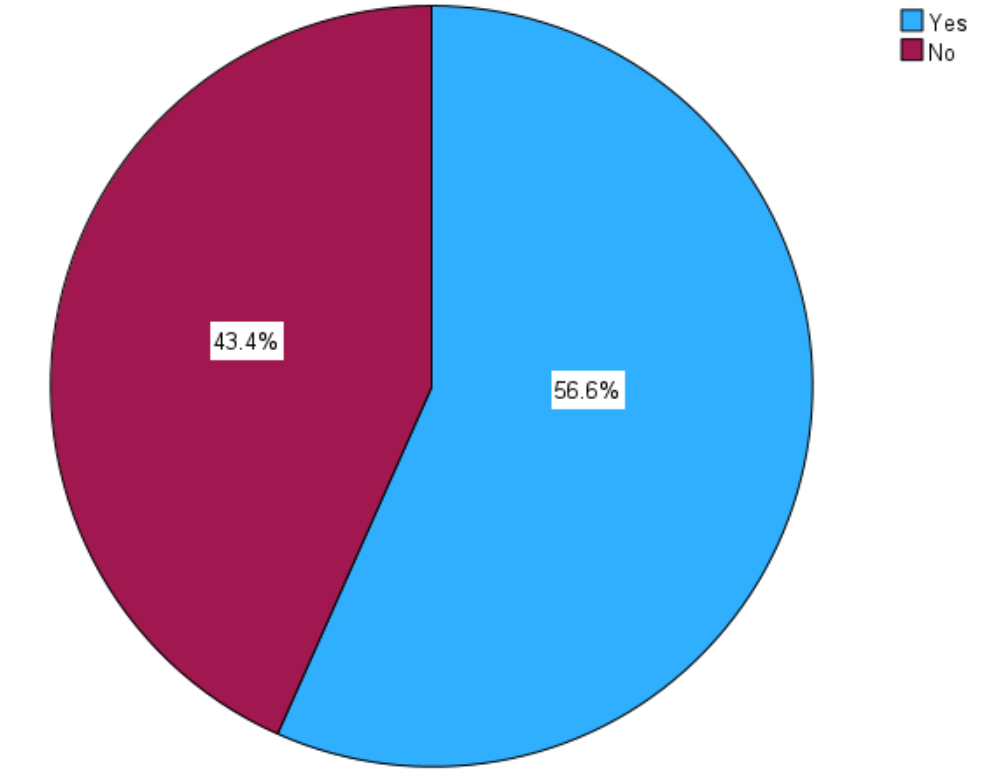 |
| Taken off Treatment  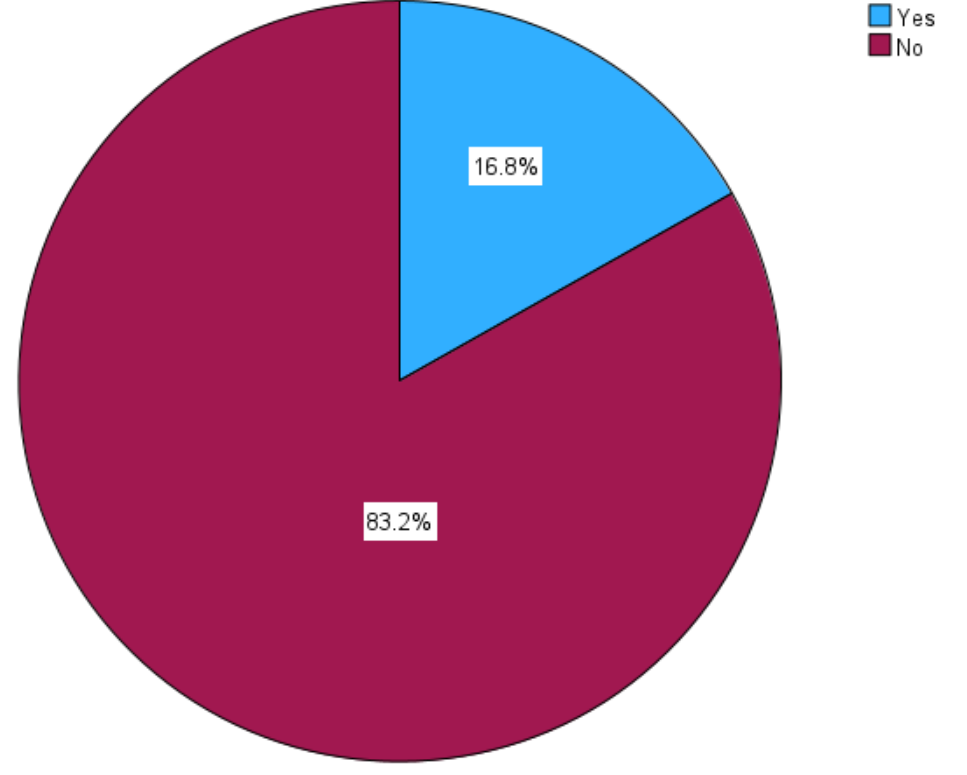 | Off Treatment Options  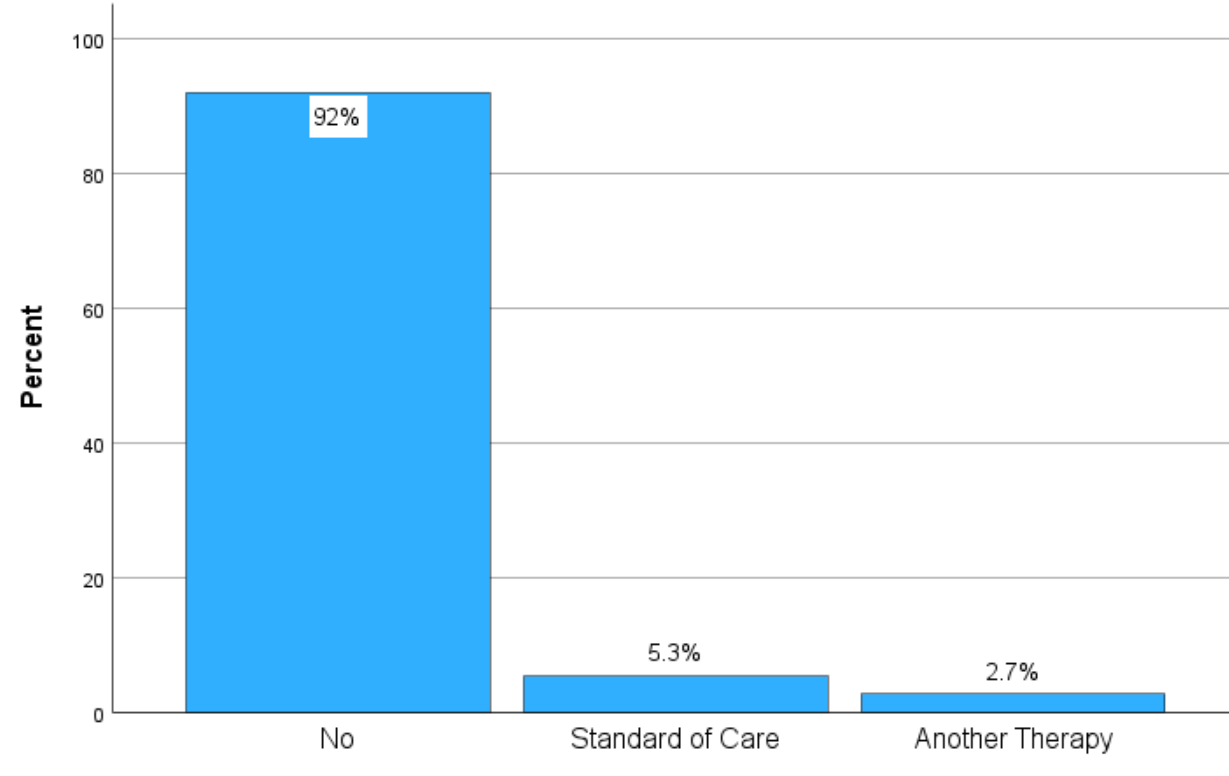 |
| Disease Progression  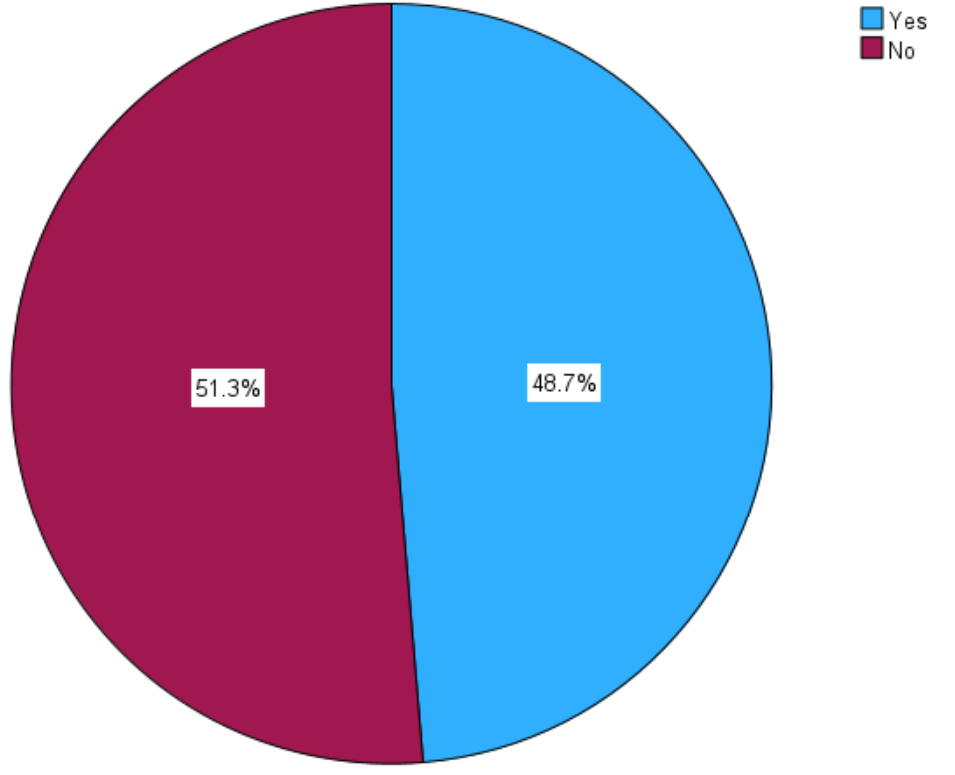 | Disease Progression Options  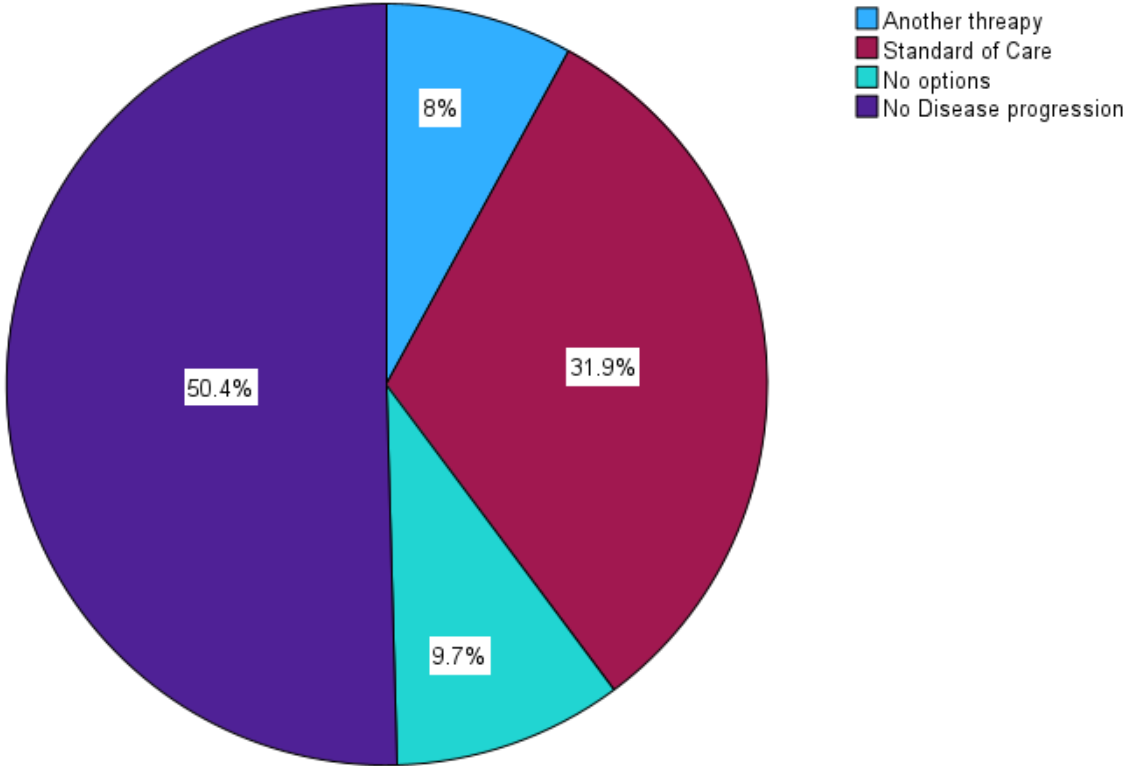 |

Figure 1. Response Frequencies, Source: SPSS

**Appendix II – Hybrid IRT Model Output**

|  | Coef. | Std. Err. | z | P>z | [95% Conf. | Interval] |
| --- | --- | --- | --- | --- | --- | --- |
| 2PL ANALYSIS |  |  |  |  |  |  |
| Rcvd_Therapy |  |  |  |  |  |  |
| Discrim | 33.7215 | 24.7621 | 1.36 | 0.173 | -14.8113 | 82.25432 |
| Diff | 0.73919 | 0.050302 | 14.7 | 0 | 0.83778 | 0.6406 |
|  |  |  |  |  |  |  |
| Offered_Tr~l |  |  |  |  |  |  |
| Discrim | 0.727631 | 0.277672 | 2.62 | 0.009 | 0.183405 | 1.271857 |
| Diff | 0.739933 | 0.35848 | 2.06 | 0.039 | 0.037326 | 1.44254 |
|  |  |  |  |  |  |  |
| Side_Effects |  |  |  |  |  |  |
| Discrim | 1.587102 | 0.398564 | 3.98 | 0 | 0.805932 | 2.368273 |
| Diff | -0.13242 | 0.168729 | -0.78 | 0.433 | -0.46312 | 0.198286 |
|  |  |  |  |  |  |  |
| Off_Treatm~t |  |  |  |  |  |  |
| Discrim | 12.22568 | . | . | . | . | . |
| Diff | 0.934464 | 0.112055 | 8.34 | 0 | 0.714839 | 1.154088 |
|  |  |  |  |  |  |  |
| Disease_Prog |  |  |  |  |  |  |
| Discrim | 0.851385 | 0.278733 | 3.05 | 0.002 | 0.305078 | 1.397692 |
| Diff | 0.135084 | 0.249109 | 0.54 | 0.588 | -0.35316 | 0.623329 |
|  |  |  |  |  |  |  |
| GPCM ANALYSIS |  |  |  |  |  |  |
| Time_Physi~n |  |  |  |  |  |  |
| Discrim | 0.780674 | 0.218741 | 3.57 | 0 | 0.35195 | 1.209398 |
| Diff |  |  |  |  |  |  |
| 2 vs 1 | 0.168371 | 0.30053 | 0.56 | 0.575 | -0.42066 | 0.7574 |
| 3 vs 2 | 1.352707 | 0.414335 | 3.26 | 0.001 | 0.540626 | 2.164788 |
|  |  |  |  |  |  |  |
| Appt_Time |  |  |  |  |  |  |
| Discrim | 1.816515 | 0.390894 | 4.65 | 0 | 1.050376 | 2.582653 |
| Diff |  |  |  |  |  |  |
| 2 vs 1 | -1.09177 | 0.209673 | -5.21 | 0 | -1.50272 | -0.68082 |
| 3 vs 2 | 1.203712 | 0.224414 | 5.36 | 0 | 0.763869 | 1.643555 |
|  |  |  |  |  |  |  |
| Appt_Count |  |  |  |  |  |  |
| Discrim | 2.713084 | 0.634588 | 4.28 | 0 | 1.469314 | 3.956854 |
| Diff |  |  |  |  |  |  |
| 2 vs 1 | -0.8843 | 0.160088 | -5.52 | 0 | -1.19807 | -0.57054 |
| 3 vs 2 | 0.663092 | 0.140794 | 4.71 | 0 | 0.38714 | 0.939043 |
|  |  |  |  |  |  |  |
| Commute |  |  |  |  |  |  |
| Discrim | 1.059352 | 0.306627 | 3.45 | 0.001 | 0.458374 | 1.66033 |
| Diff |  |  |  |  |  |  |
| 2 vs 1 | -2.51886 | 0.587369 | -4.29 | 0 | -3.67008 | -1.36763 |
| 3 vs 2 | 1.843773 | 0.464403 | 3.97 | 0 | 0.933561 | 2.753986 |
|  |  |  |  |  |  |  |
| Fin_Impact |  |  |  |  |  |  |
| Discrim | 0.16884 | 0.162864 | 1.04 | 0.3 | -0.15037 | 0.488048 |
| Diff |  |  |  |  |  |  |
| 2 vs 1 | -0.82047 | 1.413982 | -0.58 | 0.562 | -3.59182 | 1.950886 |
| 3 vs 2 | 9.06489 | 8.6881 | 1.04 | 0.297 | -7.96347 | 26.09325 |
|  |  |  |  |  |  |  |
| NRM ANALYSIS |  |  |  |  |  |  |
| Region |  |  |  |  |  |  |
| Discrim |  |  |  |  |  |  |
| 2 vs 1 | -0.23148 | 0.216309 | -1.07 | 0.285 | -0.65543 | 0.192482 |
| Diff |  |  |  |  |  |  |
| 2 vs 1 | 0.264611 | 0.84966 | 0.31 | 0.755 | -1.40069 | 1.929915 |
|  |  |  |  |  |  |  |
| Therapy_Type |  |  |  |  |  |  |
| Discrim |  |  |  |  |  |  |
| 2 vs 1 | -35.6284 | 24.64983 | -1.45 | 0.148 | -83.9412 | 12.68439 |
| 3 vs 1 | -6.48189 | 11.34975 | -0.57 | 0.568 | -28.727 | 15.76322 |
| 4 vs 1 | -2.75906 | 1.975098 | -1.4 | 0.162 | -6.63018 | 1.112064 |
| 5 vs 1 | -2.61486 | 2.067765 | -1.26 | 0.206 | -6.6676 | 1.437891 |
| 6 vs 1 | -0.53106 | 1.512526 | -0.35 | 0.726 | -3.49555 | 2.433441 |
| 7 vs 1 | -4.264 | 2.751921 | -1.55 | 0.121 | -9.65767 | 1.129665 |
| 8 vs 1 | -1.65782 | 1.516111 | -1.09 | 0.274 | -4.62934 | 1.313704 |
| Diff |  |  |  |  |  |  |
| 2 vs 1 | -0.55441 | 0.151014 | -3.67 | 0 | -0.8504 | -0.25843 |
| 3 vs 1 | -0.00594 | 0.777814 | -0.01 | 0.994 | -1.53043 | 1.51855 |
| 4 vs 1 | 0.761316 | 0.44941 | 1.69 | 0.09 | -0.11951 | 1.642145 |
| 5 vs 1 | 0.79553 | 0.494647 | 1.61 | 0.108 | -0.17396 | 1.76502 |
| 6 vs 1 | 4.889003 | 11.49228 | 0.43 | 0.671 | -17.6354 | 27.41345 |
| 7 vs 1 | 0.478908 | 0.430841 | 1.11 | 0.266 | -0.36553 | 1.323341 |
| 8 vs 1 | 2.700559 | 1.74997 | 1.54 | 0.123 | -0.72932 | 6.130438 |
|  |  |  |  |  |  |  |
| Motivation |  |  |  |  |  |  |
| Discrim |  |  |  |  |  |  |
| 2 vs 1 | -0.06222 | 0.244904 | -0.25 | 0.799 | -0.54222 | 0.417786 |
| 3 vs 1 | 0.044773 | 0.26744 | 0.17 | 0.867 | -0.4794 | 0.568945 |
| 4 vs 1 | 0.438431 | 0.688504 | 0.64 | 0.524 | -0.91101 | 1.787875 |
| Diff |  |  |  |  |  |  |
| 2 vs 1 | -8.32399 | 32.90084 | -0.25 | 0.8 | -72.8085 | 56.16047 |
| 3 vs 1 | 16.39965 | 97.82086 | 0.17 | 0.867 | -175.326 | 208.125 |
| 4 vs 1 | 5.552246 | 8.216335 | 0.68 | 0.499 | -10.5515 | 21.65597 |
|  |  |  |  |  |  |  |
| App_Dislike |  |  |  |  |  |  |
| Discrim |  |  |  |  |  |  |
| 2 vs 1 | 0.843285 | 0.296641 | 2.84 | 0.004 | 0.26188 | 1.424689 |
| 3 vs 1 | 0.76483 | 0.295993 | 2.58 | 0.01 | 0.184694 | 1.344966 |
| Diff |  |  |  |  |  |  |
| 2 vs 1 | -0.76035 | 0.382903 | -1.99 | 0.047 | -1.51083 | -0.00987 |
| 3 vs 1 | -0.67776 | 0.405997 | -1.67 | 0.095 | -1.4735 | 0.11798 |
|  |  |  |  |  |  |  |
| Off_Treat_~s |  |  |  |  |  |  |
| Discrim |  |  |  |  |  |  |
| 2 vs 1 | 14.5399 | 2.114629 | 6.88 | 0 | 10.3953 | 18.68449 |
| 3 vs 1 | 15.55844 | 9.423287 | 1.65 | 0.099 | -2.91086 | 34.02775 |
| Diff |  |  |  |  |  |  |
| 2 vs 1 | 1.198963 | 0.174373 | 6.88 | 0 | 0.857199 | 1.540728 |
| 3 vs 1 | 1.261029 | 0.226378 | 5.57 | 0 | 0.817336 | 1.704722 |
|  |  |  |  |  |  |  |
| Progress_T~t |  |  |  |  |  |  |
| Discrim |  |  |  |  |  |  |
| 2 vs 1 | -1.17374 | 0.751322 | -1.56 | 0.118 | -2.64631 | 0.29882 |
| 3 vs 1 | -0.23811 | 0.842367 | -0.28 | 0.777 | -1.88912 | 1.412903 |
| 4 vs 1 | -1.78155 | 0.765755 | -2.33 | 0.02 | -3.2824 | -0.2807 |
| Diff |  |  |  |  |  |  |
| 2 vs 1 | 1.703714 | 0.752037 | 2.27 | 0.023 | 0.229748 | 3.177681 |
| 3 vs 1 | 1.59694 | 3.601336 | 0.44 | 0.657 | -5.46155 | 8.655429 |
| 4 vs 1 | 1.365885 | 0.404821 | 3.37 | 0.001 | 0.572449 | 2.15932 |
